# Strategies to minimize false positives and interpret novel microdeletions based on maternal copy-number variants in 87,000 noninvasive prenatal screens

**DOI:** 10.1186/s12920-018-0410-6

**Published:** 2018-10-19

**Authors:** Kristjan Eerik Kaseniit, Gregory J Hogan, Kevin M D’Auria, Carrie Haverty, Dale Muzzey

**Affiliations:** Myriad Women’s Health (previously Counsyl), 180 Kimball Way, South San Francisco, CA 94080 USA

**Keywords:** Noninvasive prenatal screening, Copy-number variant, Microdeletion, Variant interpretation

## Abstract

**Background:**

Noninvasive prenatal screening (NIPS) of common aneuploidies using cell-free DNA from maternal plasma is part of routine prenatal care and is widely used in both high-risk and low-risk patient populations. High specificity is needed for clinically acceptable positive predictive values. Maternal copy-number variants (mCNVs) have been reported as a source of false-positive aneuploidy results that compromises specificity.

**Methods:**

We surveyed the mCNV landscape in 87,255 patients undergoing NIPS. We evaluated both previously reported and novel algorithmic strategies for mitigating the effects of mCNVs on the screen’s specificity. Further, we analyzed the frequency, length, and positional distribution of CNVs in our large dataset to investigate the curation of novel fetal microdeletions, which can be identified by NIPS but are challenging to interpret clinically.

**Results:**

mCNVs are common, with 65% of expecting mothers harboring an autosomal CNV spanning more than 200 kb, underscoring the need for robust NIPS analysis strategies. By analyzing empirical and simulated data, we found that general, outlier-robust strategies reduce the rate of mCNV-caused false positives but not as appreciably as algorithms specifically designed to account for mCNVs. We demonstrate that large-scale tabulation of CNVs identified via routine NIPS could be clinically useful: together with the gene density of a putative microdeletion region, we show that the region’s relative tolerance to duplications versus deletions may aid the interpretation of microdeletion pathogenicity.

**Conclusions:**

Our study thoroughly investigates a common source of NIPS false positives and demonstrates how to bypass its corrupting effects. Our findings offer insight into the interpretation of NIPS results and inform the design of NIPS algorithms suitable for use in screening in the general obstetric population.

**Electronic supplementary material:**

The online version of this article (10.1186/s12920-018-0410-6) contains supplementary material, which is available to authorized users.

## Background

Noninvasive prenatal screening (NIPS) aims to detect fetal chromosomal abnormalities early in pregnancy by quantifying cell-free DNA (cfDNA) in maternal plasma [1]. Due to its high sensitivity and specificity, clinical ease, low cost, and minimal risk of complications, NIPS has been widely adopted for the general obstetric population, including high- and average-risk pregnancies [2]. High specificity is critical in fetal aneuploidy screening, because professional guidelines recommend that all patients with positive aneuploidy results be offered follow-up invasive testing [2, 3], a procedure associated with an increased risk of pregnancy loss [4].

When performing NIPS by whole genome sequencing (WGS) of cfDNA, a sample is considered aneuploid for a given region if it has a statistically significant deviation in the number of sequenced fragments (“depth”) relative to the average depth of disomic background samples and/or regions. Because most cfDNA originates from the mother, copy-number variants in the maternal genome (mCNVs) can cause sufficiently large depth deviations to yield false positives, thereby reducing the specificity of NIPS. Indeed, the depth deviation of an mCNV relative to a fetal anomaly is so strong that even small mCNVs can have a large impact on specificity; mCNVs spanning ≥250 kb were predicted to increase the false-positive rate by 40- to 1000-fold or more [5]. Further, two recent studies of trisomies 13, 18, and 21 attributed one-third to one-half of NIPS false positives to maternal duplications [6, 7]. A 22-study meta-analysis of NIPS discordances found that 48% of false positives with an identified cause were due to mCNVs [8]. These findings underscore the need for NIPS bioinformatics pipelines to be robust to these confounding variants.

**Fig. 1:**
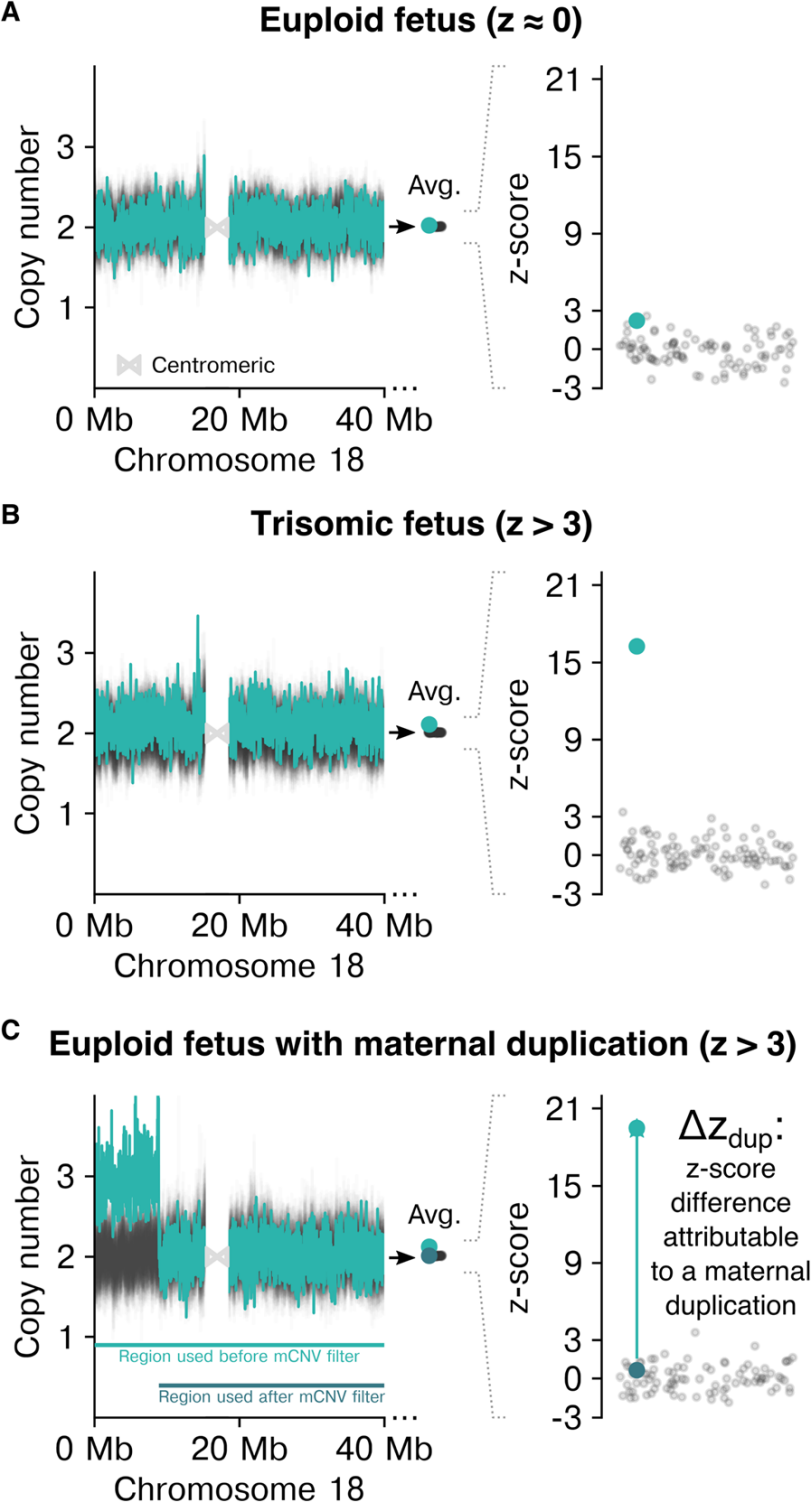
Isolating the effect on z-scores of mCNVs, a common source of NIPS false positives. For euploid (**a**), trisomic (**b**), and mCNV-harboring (**c**) samples on chromosome 18, the copy-number values in tiled 20 kb bins (see Methods) shown at left for the sample of interest (teal) and background samples (black). Shown in the middle of each panel is the average copy-number across all bins, which contributes to the z-score distribution shown at right. In (**c**), the average and z-score are calculated in the presence and absence of the mCNV; the mCNV-specific z-score gain defines *∆z*_*dup*_

A z-score is a common statistic used in WGS-based NIPS to describe the deviation of observed from expected depth values, with a higher z-score indicating a gain in DNA suggestive of a fetal trisomy (Fig. 1a, b). The depth of a region of interest (e.g., chromosome or microdeletion) is typically measured by first subdividing the region into non-overlapping bins of equal size (e.g., 20 kb) and then calculating the average depth per bin [9]. As opposed to simply calculating a region’s average depth by dividing the total mapped sequenced fragments (“reads”) by its length, an average across bins provides a straightforward way to detect and omit localized anomalies such as mCNVs and alignment artifacts. If not appropriately mitigated, mCNVs cause false aneuploid calls (Fig. 1c) because they strongly deflect the depth in their encompassing bins, and this deviation affects the average bin depth and resulting z-score in a region of interest.

In addition to enhancing the search for mCNVs, partitioning reads into bins also facilitates the identification of subchromosomal fetal CNVs like microdeletions. The aneuploidy-detection algorithm can enumerate each sufficiently lengthy set of contiguous bins as a possible microdeletion, evaluate an average, compute a z-score, and yield an assertion of fetal copy number. Recent studies have shown that WGS-based NIPS data reveal novel fetal CNVs at a resolution of 7 Mb [10]; however, the clinical interpretation of such variants is not straightforward, and the utility of reporting them to patients is unestablished.

We sought to explore the impact of mCNVs on the identification and interpretation of fetal chromosomal abnormalities. Our first step was to develop an mCNV-finding algorithm to measure the frequency of mCNVs and identify patterns in their genomic locations. Next, we evaluated the impact of mCNVs on NIPS specificity, highlighting the virtues and drawbacks of different algorithmic strategies, including both adapted and novel approaches. Finally, we used the observed frequency, length, and positional distribution of mCNVs—coupled with the assumption that most mCNVs are benign—to shed light on the clinical interpretation of novel fetal microdeletions.

## Methods

### Analysis of NIPS samples

The protocol for this study was reviewed and designated as exempt by Western Institutional Review Board and complied with the Health Insurance Portability and Accountability Act (HIPAA). The information associated with patient samples was de-identified in accordance with the HIPAA Privacy Rule. A waiver of informed consent was requested and approved by the IRB. A total of 87,255 de-identified samples meeting internal quality control criteria were retrospectively analyzed for the presence of mCNVs across all chromosomes. Samples without mCNVs and fetal aneuploidies comprised a subset later employed for mCNV simulations (described below).

### mCNV detection

mCNVs were detected using a moving-window approach that considered copy-number values in 20 kb bins tiling each chromosome. A bin’s copy-number value is a fractional number (e.g., 1.997) that reflects the bin’s read depth and results from multiple normalization steps described below in the section about mCNV handling. The presence or absence of an mCNV was assessed at each bin *i*. First, the median copy-number value across the 10 bins *i* through *i* + *9* was calculated in both the sample of interest and in background samples. A z-score was computed for each sample’s observed median copy-number value relative to the background average. Bins *i* through *i* + *9* were classified as part of an mCNV if (1) the absolute median copy-number value was <1.5 or >2.5, and (2) the absolute z-score was determined to be significant. As some genomic bins are filtered out elsewhere in the analysis pipeline (e.g., for spuriously high read depth or for “unmappable” regions with redundant sequences that complicate unique mapping of reads), gaps of up to five genomic bins within mCNVs were allowed. Consecutive mCNV calls of the same type were merged if the resulting call had a significant z-score. For example a 12-bin mCNV would be called by merging three mCNV calls starting at bins *i*, *i* + *1* and *i* + *2*, or a 25-bin call could be made by merging calls starting at bins *i* and *i* + *15* (if bins *i* + *10* through *i* + *14* were a gap). The edges of merged calls were trimmed by up to 10 bins on either side, with the final mCNV boundaries determined by the pair of edges that maximized the absolute z-score of the call. Due to the trimming, calls smaller than 200 kb were possible if the trimmed set of bins yielded a large enough absolute z-score. Aside from this section, z-score refers to the aneuploidy z-score, not the z-score of the mCNV. Additional file 1: Figures S1 and S2 illustrate the efficacy of this mCNV-detection algorithm on simulated samples, which are themselves described further below.

### Strategies for mCNV handling

For six NIPS bioinformatic analysis pipelines, we evaluated the specificity of whole-chromosome aneuploidies as a function of the presence of mCNVs. Each pipeline differed in key ways as described below but shared a common general analysis foundation: mapping short NGS reads from WGS of cfDNA to a reference genome, counting the number of reads per genomic bin (20 kb), applying GC-content corrections at the read level [11] and mappability corrections at the bin level [12], normalizing these reads-per-bin values at the sample and bin level, calculating an average of these values per chromosome, and comparing the sample-specific averages of the chromosome to the averages of background samples using a z-score. The z-score is calculated based on measures of central tendency (e.g., mean or median) and dispersion (e.g., standard deviation). Each approach below differs in how these measures are calculated. The left panels of Fig. 4 illustrate the mechanics of each strategy.

The first pipeline, “Simple,” is based on the initially published algorithms for NIPS [13] and does not feature any mCNV-specific nor generally robust features. The method calculates z-scores using the mean and standard deviation of the bin copy-number values without any outlier filtering.

The second pipeline, “Robust,” builds on the “Simple” method, uses the median in place of the mean, and estimates the standard deviation by (1) calculating the interquartile range (IQR) of bin copy-number values, and (2) converting the IQR to an estimate of standard deviation based on the assumption that the data are normally distributed [14]. Algorithms that use robust statistical measures in some but not all steps of the z-score calculation have been previously reported [15].

The third pipeline, “Robust+Gaussian,” refines the central tendency and dispersion estimations by (1) discarding the top and bottom fifth percentiles of the region’s copy-number values, (2) fitting a Gaussian function to the copy-number values of a region, and (3) discarding any values more than four standard deviations away from the estimated mean. Similar methods of discarding outlying bins—without explicit mCNV detection—have been reported previously [7].

The fourth pipeline, “Z-correction,” is inspired by a previously proposed compensation approach [16]. The approach assumes that mCNVs have a consistent, size-specific effect on aneuploidy z-scores and corrects for this. Our implementation uses results from the “Robust” pipeline but subtracts a z-score offset for chromosomes harboring an mCNV that is itself a function of the mCNV size. The mapping of mCNV size to z-score offset was determined via simulations (described below).

The fifth pipeline, “Value filtering,” builds upon the “Robust” pipeline by filtering out any bins with copy-number value less than *c*_*low*_ = 1.5 or more than *c*_*high*_ = 2.5. The cutoff pair *c*_*low*_ = 1.61 and *c*_*high*_ = 2.35 based on the empirical bin copy-number value distribution values within and outside of mCNVs (Additional file 1: Figure S3 and S4) was also analyzed.

The sixth pipeline, “mCNV filtering,” builds upon the “Robust” pipeline by identifying mCNVs and ignoring their constituent genomic bins on an individualized, per-sample basis when calculating the central tendency and dispersion.

Additional file 1: Table S1 summarizes the various algorithm strategies considered.

### mCNV simulations

To supplement the mCNVs observed in our patient cohort and characterize algorithm performance for arbitrary mCNV sizes, we simulated mCNVs by scaling the bin-level copy-number values obtained from patient samples. We focused our analysis on maternal duplications as they can lead to false positives in the analysis of trisomies. For the region in which we wanted to simulate a CNV, the copy-number values were multiplied by a factor that mimics the gain observed in empirical maternal duplications; the expected ratio of bin copy numbers in maternal duplications vs. non-mCNV regions is 3/2 = 1.50, but we observed this factor to be slightly lower at 2.88/2 = 1.44 (Additional file 1: Figure S3). This approach further assumes that simulated mCNVs were inherited by the fetus. mCNVs not inherited by the fetus would have marginally decreased signal in proportion to the fetal fraction, and this would reduce their potentially compromising effect on specificity but also make them slightly more difficult to detect.

For each of the chromosomes 13, 18, and 21, at least 10,000 mCNV-harboring samples were simulated, each using as a baseline a randomly chosen sample shown to be both euploid (via the “mCNV filtering” pipeline) and void of mCNVs. Most samples (83%) were chosen for exactly one round of simulation, with the rest used in several rounds of simulations (15% in two and 2% in 3 or more simulations). The sizes of the mCNVs were selected to span a logarithmic range, and the position of each mCNV was randomly chosen. The mCNV size values used in downstream analyses were based on the simulated boundaries rather than the algorithm-detected boundaries (e.g., a 3 Mb simulated duplication identified as being 2.8 Mb by the mCNV-finding algorithm is represented in the plots and associated analyses herein based on the 3 Mb size; Additional file 1: Figure S1).

### Maternal duplication impact analysis

The impact of maternal duplications on aneuploidy z-scores was evaluated in both empirical and simulated samples.

The empirical approach included only those samples observed to have an mCNV, and it estimated the median aneuploidy z-score as a function of the duplication size. If a chromosome contained multiple mCNVs, the duplication size was the sum of the observed mCNV lengths. The aneuploidy z-score has an expectation of 0 for euploid samples, and the median is not expected to deviate appreciably from 0 even if some trisomic samples are present due to their relative rarity. Hence, a systematic positive shift of the median z-score as a function of maternal duplication size is consistent with mCNVs underlying some NIPS false positives.

The simulation-based approach directly estimated the effect of maternal duplications on z-scores and, subsequently, on specificity. We defined *∆z*_*dup*_ = *z*_*mCNV+*_ − *z*_*mCNV−*_ as the z-score difference attributable to a maternal duplication (Fig. 1c), with *z*_*mCNV+*_ and *z*_*mCNV−*_, respectively, representing the z-score with and without the simulated mCNV. For a given size of mCNV, positive *∆z*_*dup*_ values indicate z-scores are sensitive to the presence of maternal duplications, and no shift (*∆z*_*dup*_ of 0) means the bioinformatic analysis pipeline is not biased by mCNVs.

To calculate the specificity of NIPS as a function of mCNV size, we modeled the z-score of a euploid sample harboring an mCNV as a random variable *Z* = *Z*_*mCNV−*_ + *∆Z*_*dup*_. *Z*_*mCNV−*_ represents the z-score of a sample without an mCNV. It follows a standard normal distribution N(*μ* = 0, *σ* = 1) and is not a function of mCNV size. By contrast, for an mCNV of size *s*, *∆Z*_*dup*_ is normally distributed with mean *μ*_*dup*_ and standard deviation *σ*_*dup*_ calculated from the *∆z*_*dup*_ values of the 200 simulated samples whose mCNV sizes were closest to *s*. Assuming *Z*_*mCNV−*_ and *∆Z*_*dup*_ are independent, *Z* is a normal random variable with mean *μ*_*dup*_ and standard deviation (1 + *σ*_*dup*_^2^)^0.5^. Since the simulations introduced mCNVs into otherwise euploid samples, any modeled positives (i.e., *Z* = *Z*_*mCNV−*_ + *∆Z*_*dup*_ > 3) were false positives. Furthermore, any modeled samples with *z*_*mCNV−*_ > 3 were considered to be statistical false positives. Hence, the false-positive rate (FPR) attributable to mCNVs was calculated by omitting these statistical false positives:

$$ {FPR}_{mCNV}=\mathrm{P}\left({Z}_{mCNV-}+{\varDelta Z}_{dup}>3\right)-\mathrm{P}\left({Z}_{mCNV-}>3\right) $$

Specificity was simply *1* − *FPR*_*mCNV*_. The specificity as a function of mCNV size was estimated for each chromosome separately using simulated samples with mCNVs introduced on the chromosome of interest.

**Fig. 2:**
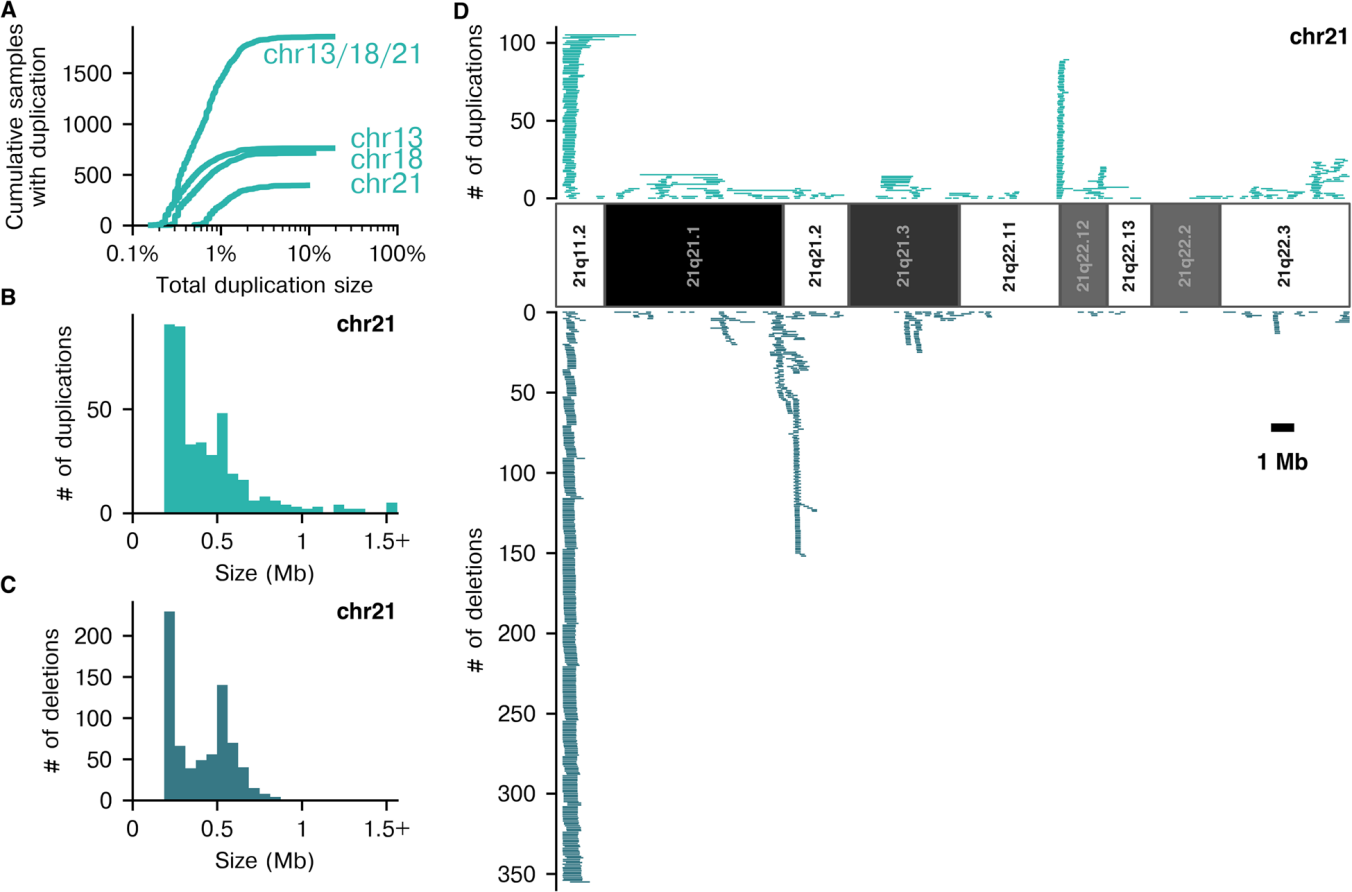
mCNVs vary in positional frequency and length. **a** Cumulative distribution of mCNV duplication size on the three commonly aneuploid autosomes—as well as their aggregate (“chr13/18/21”)—expressed as the percentage of the chromosome the mCNV spans (*N* = 87,255 samples). **b**, **c** The size distribution of mCNVs on chromosome 21 for duplications (**b**) and deletions (**c**). **d** For the mappable regions of chromosome 21, lines indicate observed mCNV positions and lengths (1 Mb scale bar indicated) for duplications (top) and deletions (bottom)

The estimate of cumulative false positives due to mCNVs per 100,000 was calculated as the weighted sum of the empirical maternal-duplication size-prevalence data (Fig. 2b) multiplied by the size-dependent specificity data from the simulation-based analysis (Fig. 4, right column).

## Results

### Autosomal mCNVs larger than 200 kb are detected in 65% of patients and cover the majority of the genome

As a first step toward measuring the impact of mCNVs on NIPS performance, we surveyed their frequency, size, and positional bias in 87,255 patient samples. Using a rolling-window z-score algorithm (see Methods), we identified mCNVs ≥200 kb. On average, patients had 1.07 autosomal mCNVs, and 65% of patients had at least one mCNV. There were 37% more deletions than duplications overall, but duplications were generally larger than deletions (median sizes 360 and 260 kb, respectively; Kruskal-Wallis H-test *p* < 0.05).

Chromosomes 13, 18, and 21 are commonly tested in NIPS, and mCNVs on these chromosomes pose the most direct risk for false positives. On these chromosomes, 2.1% of all patients had at least one duplication and 2.5% had at least one deletion with 4.5% having an mCNV of either type (Fig. 2a). On chromosome 21, deletions and duplications were observed at a similar frequency, yet mCNVs larger than 1 Mb were all duplications (21 duplications and no deletions, Fig. 2b, c). The high frequency of mCNVs on the commonly trisomic chromosomes illustrates why an NIPS strategy that results in no-calls for samples with mCNVs would be clinically inviable, as the rate of no-calls and invasive follow-up procedures would be unacceptably frequent.

We investigated the positional distribution of mCNVs to evaluate the previously published premise [13] that if mCNV positions were highly predictable, an algorithm could achieve robustness simply by masking out (or “blacklisting”) such regions. Indeed, we observed that mCNVs were not distributed uniformly (Fig. 2d). Hotspots of mCNVs were common, with some hotspots having an equal number of duplications and deletions, and others having an imbalanced ratio of the two. However, mCNVs were not constrained to hotspot regions, as they were observed across nearly all of the mappable portion of chromosome 21, with only about 14% of the chromosome having no observed mCNVs in our dataset (approximately 7% of chromosome 13 and 9% of chromosome 18 did not have mCNVs; Additional file 1: Figure S5). Though mCNV hotspots suggest that a blacklist approach could partially mitigate the impact of mCNVs, this strategy has drawbacks: either (1) many sites are blacklisted, which would impair sensitivity for aneuploidy detection or (2) few sites are blacklisted, after which many samples would retain mCNVs within the analyzed regions that could lower specificity. This result extends to NIPS assays that apply the blacklist at a biochemical level, e.g., by only targeting certain regions for sequencing [17, 18].

### The impact of mCNVs on z-scores observed in empirical data is recapitulated and supplemented with simulations

**Fig. 3:**
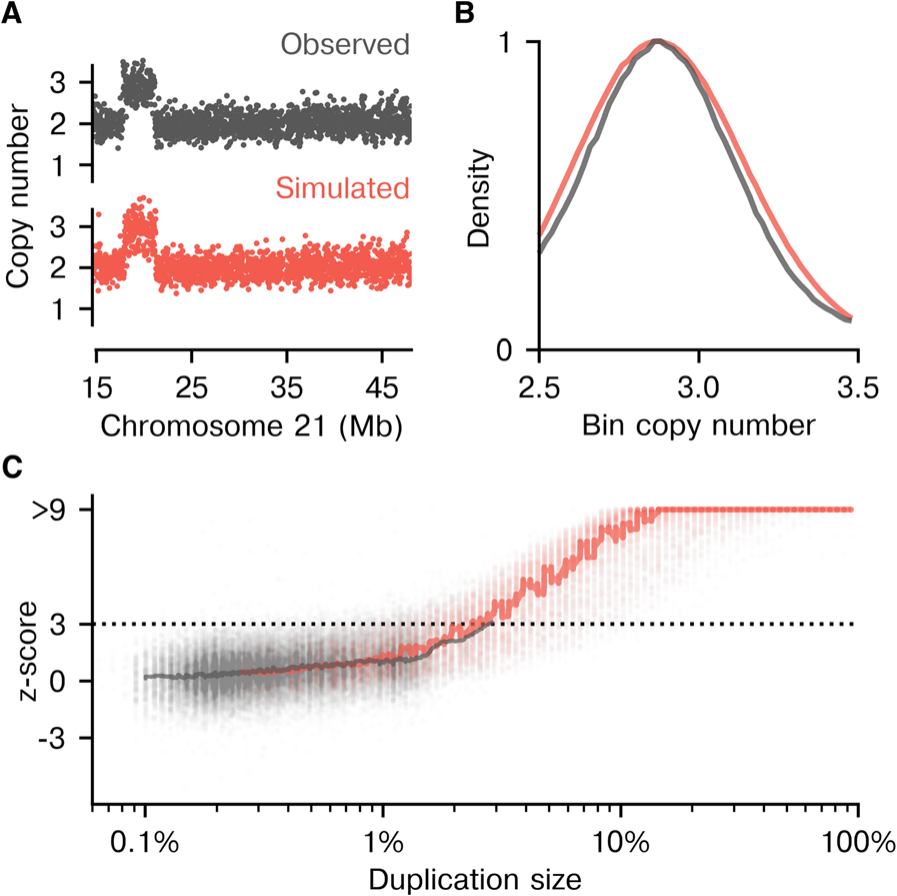
Simulating mCNVs enables thorough performance analysis. **a** Simulated bin-level copy-number trace for a simulated sample containing an mCNV on chr21 (red) is highly similar to the trace for an observed sample (gray) with a similar maternal variant. **b** The probability distribution of copy-number values for bins within mCNVs is similar for simulated (red) and observed (gray) samples. **c** There is a similar upward trend in z-scores for observed (gray; *N* = 38,102 data points from 87,255 samples) and simulated (red; *N* = 30,887 data points, one per simulation) samples that have a maternal duplication of the indicated size on autosomes (“Simple” method). The solid line is a rolling median of 500 adjacent data points. Z-scores are capped at 9 in the plot only for visualization purposes

We next explored the impact of mCNVs on aneuploidy-calling fidelity as a function of mCNV size (Fig. 3). Empirically observed mCNVs rarely spanned ≥1% of a chromosome, which prohibited a statistically powered assessment of the impact of these large mCNVs. To overcome the sparsity of empirical data, we implemented simulations to systematically analyze the effects of maternal duplications on trisomy detection. To create a simulated sample harboring an mCNV of a given size and position, the bin-level copy-number data corresponding to the region of interest was scaled by an empirically derived factor in a euploid and mCNV-free sample (Fig. 3a, b). Simulated samples strongly resembled their observed counterparts, both at the level of bin profile (Fig. 3a) and the distribution of bin copy-number values (Fig. 3b). The bin copy number within simulated mCNVs was very slightly overdispersed compared to the bin copy numbers within detected patient mCNVs (Fig. 3b). The strong overlap between median z-scores for the empirical and simulated samples (Fig. 3c, thick gray and red lines, both for the “Simple” method) suggests that this dilation effect has a negligible impact on our results.

Maternal duplications exert an upward pressure on z-scores, and this effect was reproduced in our simulated data on autosomes (Fig. 3c, gray and red traces, respectively). Importantly, with the simulated data the effect was more readily observed, as the full size spectrum of potential mCNVs was modeled. Larger simulated duplications led to increasing positive shifts away from the expected median z-score of 0 for a euploid sample (Fig. 3c, red trace). The threat to the clinical performance of NIPS is that this bias toward higher z-scores contributes to false positives and lowers specificity. Indeed, the simulations suggest that the average sample harboring an mCNV spanning 2.4% or more of a chromosome would be expected to yield a false positive using the “Simple” approach (i.e., the median z-score exceeds 3).

### mCNV impact on z-scores can be reduced, but not eliminated, with outlier-robust algorithms

We sought to determine which algorithmic features in an NIPS analysis pipeline minimize the effect of mCNVs on z-scores. Our simulated samples were an ideal data set for this analysis, as the samples have both a “pre-mCNV” z-score (reflecting their original status as both euploid and free of mCNVs; see Methods) and a “post-mCNV” z-score calculated after introducing a modeled maternal duplication. The difference between the post- and pre-mCNV z-scores—which we term *∆z*_*dup*_—is a direct measure of the effect of mCNVs on z-scores. A positive *∆z*_*dup*_ means the aneuploidy z-score was increased with the introduction of a simulated mCNV.

**Fig. 4:**
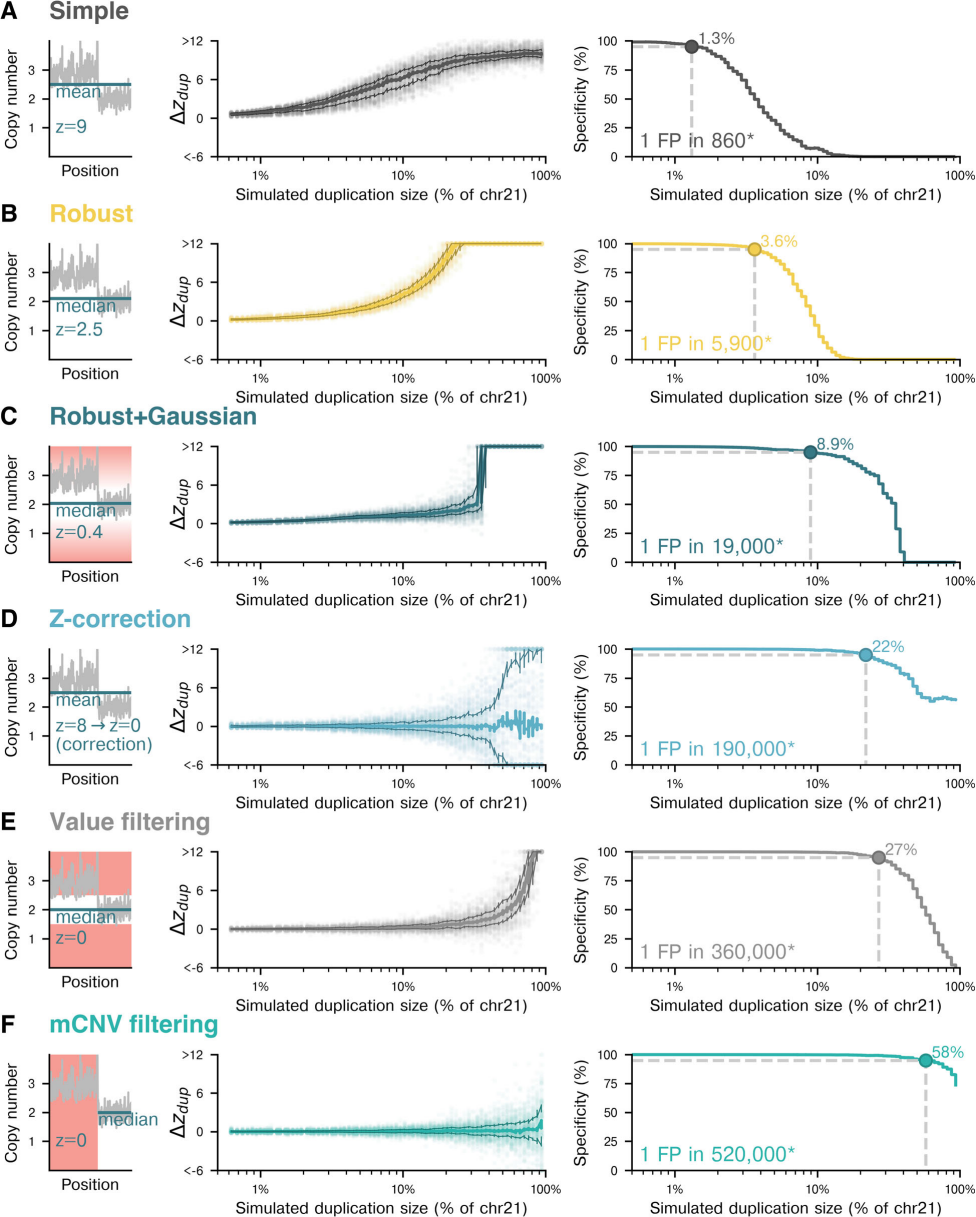
Change in z-score and specificity due to simulated maternal duplications for different analysis approaches. Each row displays the performance of the indicated analysis strategy. The left column plots *∆z*_*dup*_ as a function of maternal duplication size on chr21, with each plot having >10,000 simulated samples. The thick trace is a rolling median, and the top and bottom thinner lines are the 75th and 25th percentiles, respectively. In the right column, the impact on specificity of maternal duplications is shown, with the dashed lines indicating the position of 95% specificity for chr21. The calculations for specificity and the expected frequency of false positives are described in Methods. (*) The indicated false-positive rate is an aggregate measure based on the specificity and mCNV prevalence of chromosomes 13, 18, and 21. **a** Simple, **b** Robust, **c** Robust+Gaussian, **d** Z-correction, **e** Value filtering, **f** mCNV filtering

Six analysis strategies were tested on simulated samples with maternal duplications on chromosomes 21 (Fig. 4), 13 (Additional file 1: Figure S6), or 18 (Additional file 1: Figure S7). For each test of a strategy and a chromosome, we evaluated at least 10,000 simulated samples. As described in Methods and summarized in Additional file 1: Table S1, the strategies differ both in their approaches for calculating the central tendency (e.g., mean or median) and dispersion of bin copy-number values across a chromosome and in their filtering methods that determine which bins are used in those calculations. For each method, *∆z*_*dup*_ was plotted as a function of mCNV size (Fig. 4, middle panels), and these data were sampled to estimate how specificity falls as mCNVs grow (Fig. 4, right panels; see Methods).

The “Simple” approach (Fig. 4a) summarizes the bin copy-number values of a chromosome by the mean and standard deviation, without applying any mCNV-specific or nonspecific filters. As anticipated, this method was the most susceptible to false positives due to mCNVs; at the point where duplication size exceeded 1.3% of chromosome 21 (0.42 Mb, autosomal duplications of this size or greater observed in 13% of patients), the estimated specificity dropped below 95%, and duplications spanning more than approximately 10% of the chromosome always caused false positive results [3]. Methods using an alternative to the z-score while still using the mean and standard deviation in the analysis—such as employing a t-test [19]—would likely be similarly susceptible to mCNVs.

The “Robust” approach (Fig. 4b) improves upon the “Simple” strategy by replacing the mean with the median and estimating the standard deviation of bin copy-number values from their interquartile range, rather than calculating the standard deviation directly. The median and IQR are less susceptible to outlying bins than the mean and standard deviation; therefore, utilizing these values is expected to increase robustness to mCNVs. Indeed, this approach had smaller z-score deflections than the “Simple” strategy for mCNVs spanning <10% of the chromosome but was still suboptimal; specificity dropped below 95% for mCNVs spanning ≥3.6% (1.2 Mb) of chromosome 21, and our patient cohort contained 1168 samples (1.3%) with duplications in that size range (Fig. 2a).

The “Robust+Gaussian” approach (Fig. 4c) adds another layer of nonspecific outlier removal to the “Robust” approach by rejecting bins that fall far outside of a Gaussian fit to the bin copy-number data. This method performed better than both the “Simple” and “Robust” methods, but was susceptible to mCNVs spanning approximately 8.9% of chromosome 21 (2.9 Mb), at which point specificity dropped below 95%. As a consequence of more stringent filtering, the Robust+Gaussian method discards more bins relative to the previous strategies. This excess bin culling would reduce sensitivity because sensitivity of WGS-based NIPS is an increasing function of the number of bins [20].

### Directly accounting for mCNVs boosts specificity

We next considered strategies that specifically address mCNVs, positing that directed approaches would further boost specificity. The “Z-correction” method (Fig. 4d) first calculates a z-score for the chromosome—without removal of mCNV bins—and next subtracts a chromosome- and size-specific z-score offset determined via simulated samples analyzed with the “Robust” approach. In adjusting for mCNVs, this method assumes that the effect of mCNVs on z-score is determined by size and is reproducible across samples. This method performed better in aggregate compared to the previous approaches, as the median of *∆z*_*dup*_ remained near 0 even for large duplications. However, *∆z*_*dup*_ values were relatively highly dispersed for simulated duplications around >3% (1 Mb) in size, meaning that an mCNV would still cause large z-score deviations for some samples. The specificity for chromosome 21 dropped below 95% at duplication sizes of approximately 22% (7.0 Mb).

The “Value filtering” approach (Fig. 4e) operates on a simple premise: neutralize mCNVs by purging bins with high (>2.5) or low (<1.5) copy-number values prior to calculating the chromosome-wide average and dispersion. This method was robust to mCNVs that were not extremely large (<95% specificity for mCNVs larger than 27% of chromosome 21, or 8.7 Mb), but showed elevated variability in *∆z*_*dup*_ for all mCNV sizes relative to other strategies. The increased noise results from filtering out bins too aggressively, leaving fewer data points—and consequently more noise—for z-score calculation. Duplications are still expected to have some bins with copy-number values less than 2.5 but elevated compared to non-duplicated regions, which is likely why large duplications caused a positive *∆z*_*dup*_. A variant of this method using cutoff values based on empirical bin copy-number values is shown in Additional file 1: Figure S4. This method showed the most variability in the fraction of bins retained after filtering (Additional file 1:Figure S4, right panels) compared to all other methods that were analyzed, suggesting that it could have a nontrivial and variable impact on aneuploidy sensitivity for samples with mCNVs, as sensitivity depends on the number of bins available for z-score calculation [20].

Finally, the “mCNV filtering” approach (Fig. 4f) performs a sample-specific exclusion of bins included in mCNVs. Treating each sample separately, chromosomes are scanned for the presence of mCNVs (see Methods) and then mCNV-spanning bins are excised prior to all downstream calculations. This method was the most robust to mCNVs compared to the others, with specificity dropping below 95% only for maternal duplications larger than 58% of chromosome 21 (18 Mb). Because the “mCNV filtering” method removes only the data that should be removed, it decreases z-score noise, retains high specificity, and has more consistent sensitivity compared to the “Value filtering” approach due to less noise in the number of bins retained (Additional file 1: Figure S4, right panels).

### mCNV filtering reduces mCNV-caused false-positive rate to fewer than 1 in 520,000

To evaluate the algorithmic strategies through a more clinically relevant lens, we calculated the expected frequency of false-positive aneuploidy calls resulting from mCNVs on chromosomes 13, 18, and 21 (see Methods). Using the measured relationship between duplication size and *∆z*_*dup*_ (Fig. 3), as well as the size and chromosome of the observed maternal duplications in over 56,000 NIPS samples (the 65% of the 87,255 sample cohort with mCNVs), we estimated the false-positive rate combined across the three chromosomes for each NIPS data-analysis strategy described earlier.

On average, mCNVs are predicted to cause a false-positive result of trisomy 13, 18, or 21 for 1 in 860 patients using the “Simple” approach. This false-positive rate is similar to the rates reported by laboratories prior to incorporating changes that mitigate the effect of mCNVs: in outcome studies, Chudova et al. reported 3 mCNV-caused false positives in 1914 patients (a rate of 1 in 640) [7], and Strom et al. reported 61 mCNV-caused false positives in 31,278 patients (a rate of 1 in 510) [6]. The “Simple” estimated false-positive rate is also consistent with aggregate statistics of NIPS specificity from meta-analyses over the time period when comparable methods were common [3].

Overall, mCNV-aware approaches (“Z-correction”, “Value filtering”, “mCNV filtering”) had higher specificity than mCNV-unaware approaches. All mCNV-aware approaches increased the pooled specificity for the three common trisomies such that the aggregate false-positive rate was fewer than 1 in 100,000 tests. Remarkably, relative to the “Simple” approach with one false positive expected for every 860 samples, the “mCNV filtering” approach is expected to incur only one mCNV-caused false positive for every 520,000 samples, representing a 600-fold reduction.

### mCNVs offer insight into clinical interpretation of novel fetal microdeletions

The high frequency and positional dispersion of CNVs across the genome (Fig. 2) was noteworthy in this ostensibly healthy pregnant population. We were curious about whether the landscape of maternal copy-number variation could inform the potential clinical impact of copy-number variation in the fetal genome. Such knowledge is important because WGS-based NIPS technology can detect novel fetal microdeletions on the order of 10 Mb [10], and it is not yet clear how to interpret the health implications of such variants.

**Fig. 5:**
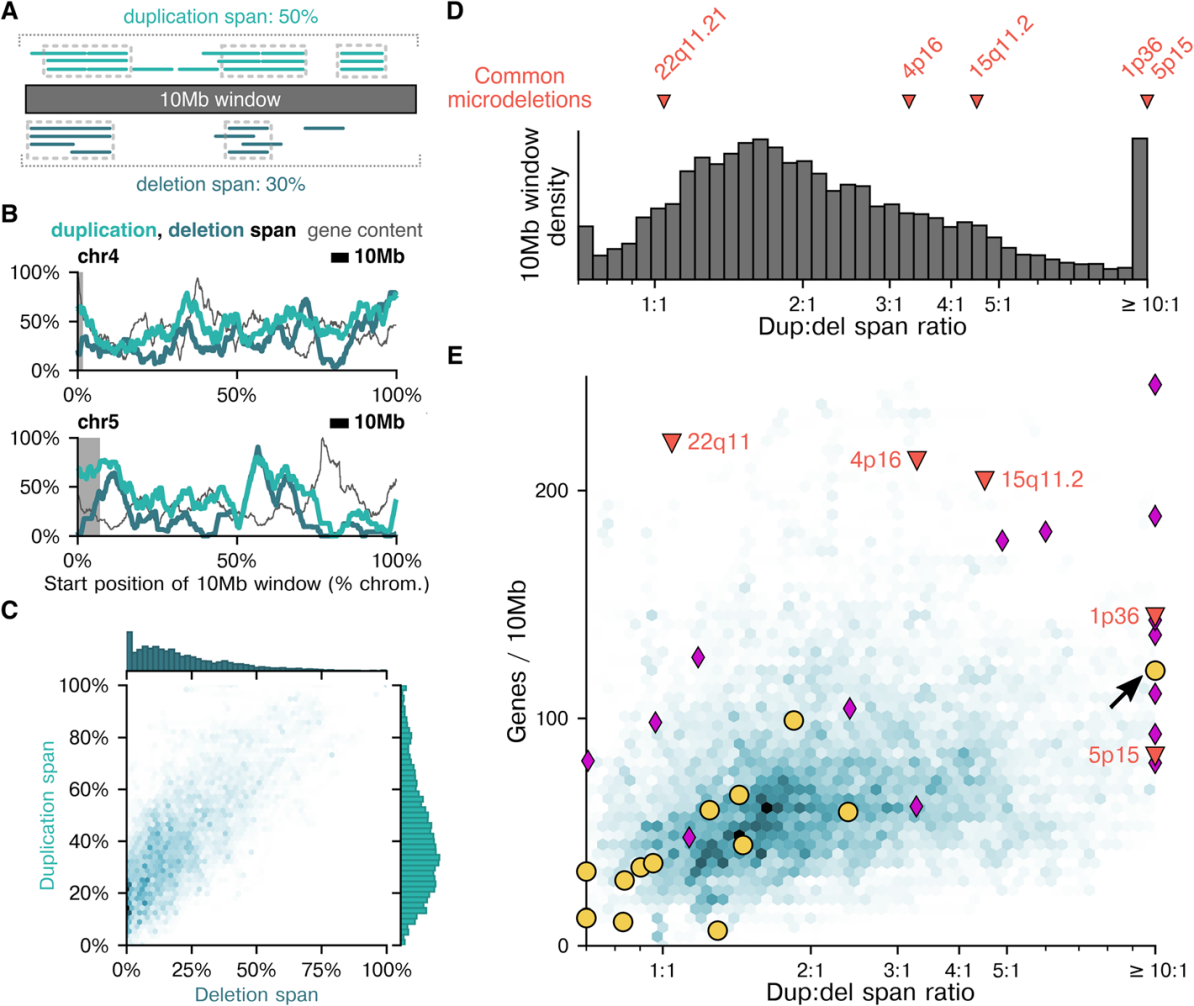
Implications of deletion prevalence in a pregnant population. **a** The “duplication span” and “deletion span” values were calculated by counting the percentage of bins in a 10 Mb window at which the depth (count) of mCNVs is ≥3. Dotted boxes demarcate regions with sufficient mCNV depth to contribute to the span percentage. In the duplication span schematic, the dotted boxes constitute 50% of bins in the 10 Mb window, and in the deletion span schematic, 30% of bins are in the dotted boxes; thus, the duplication and deletion spans 50 and 30%, respectively. **b** Examples of the span values and gene content for chromosomes 4 and 5. Gray regions indicate the common 4p16 and 5p15 microdeletions. **c** 2D histogram of the deletion and duplication spans (Pearson *r* = 0.73, *p* < 0.05) with their respective 1D histograms above and at right. **d** The dup:del ratios of common microdeletions (red triangles) are plotted relative to a histogram of dup:del ratios of 10 Mb moving windows across the autosomes. **e** Common microdeletions and most other pathogenic ICCG microdeletions (purple diamonds) are outliers in either their gene density or dup:del ratio compared to 10 Mb windows (2D histogram in background). Arrow indicates an observed pathogenic 13q34 maternal terminal microdeletion that is an outlier in both parameters, while other observed maternal deletions (yellow circles)—expected to be benign—had low values. Panels (**d**) and (**e**) plot variants with dup:del ratios outside of the shown x-axis bounds at the nearest boundary and variants with a deletion span of 0 as having a maximal dup:del ratio. **c** and **e** show 2D histograms with hexagonal bins, where dark colors are high density and light colors are low density

We reasoned that the clinical consequences of a novel 10 Mb microdeletion would be less severe if there are deletions observed throughout the region in a healthy population. Therefore, we calculated the proportion of each autosomal, 10 Mb sliding window that was covered by at least three observed deletions in our mCNV dataset, termed the “deletion span” (Fig. 5a). We assumed that duplications are more likely to be benign than deletions and, therefore, calculated the corresponding duplication span for each region to serve as a proxy to control for CNV propensity. As schematized in Fig. 5a, a window with a high duplication span has several observed duplications covering most of the region, and a window with a low deletion span has deletions only in a few parts of the region. The number of observed mCNVs in a given window is not the sole determinant of the span; for example, a 10 Mb window that had a 200 kb deletion hotspot but no deletions elsewhere would have a small deletion span. Figure 5b shows span values as a function of position across chromosomes 4 and 5 (all other chromosomes in Additional file 1: Figure S8), and Fig. 5c compares deletion and duplication spans for all 10 Mb windows across autosomes. The two span measurements were significantly correlated (Pearson *r* = 0.73, *p* < 0.05), consistent with there being an intrinsic propensity for CNVs (deletions and duplications) that varies by position [21].

Based on the presumption that deletions are more likely to be pathogenic than duplications, we expected that a small deletion span, relative to the duplication span, would be a feature of pathogenic microdeletions. Therefore, we calculated the ratio of spans (“dup:del ratio”) and evaluated whether pathogenic microdeletions had elevated dup:del ratios. Figure 5d shows the histogram of the dup:del ratio for autosomal 10 Mb bins; it highlights five commonly screened pathogenic microdeletions (22q11.21, 5p15, 1p36.32–33, 4p16.2–3, and 15q11.2–13.1). Four of the five pathogenic microdeletions had a dup:del ratio in the 75th percentile or greater, but 22q11.21 had a nearly 1:1 dup:del ratio (10th percentile). These data suggest that a high dup:del ratio could be a common—but not ubiquitous—feature of pathogenic microdeletions.

We investigated the density of genes in a region as a secondary feature that could distinguish whether a deletion is pathogenic. Notably, based on gene density and dup:del ratio, each of the common pathogenic microdeletions was an outlier relative to typical 10 Mb windows in the genome in one feature, the other, or both (a result robust to the mCNV-count threshold used to define a span, Additional file 1: Figure S9, as well as resamplings of the study population, Additional file 1: Figure S10). Microdeletion 22q11.21 had only an intermediate dup:del ratio, as mentioned, but its gene density is very high. Microdeletion 5p15, by contrast, had the opposite: an elevated dup:del ratio (≥99th percentile) but approximately average gene density. Finally, microdeletions 1p36, 4p16, and 15q11 all had both high gene density and elevated dup:del ratio.

To expand the investigation to a larger number of known pathogenic microdeletions, we additionally analyzed expert-curated pathogenic deletions [22] ≥1 Mb in length from the International Collaboration for Clinical Genomics (ICCG, formerly ISCA). Nearly all such variants were outliers in one or both metrics (purple diamonds, Fig. 5e), consistent with the findings for common microdeletions. Two known pathogenic microdeletions (2p15p16.1 and 12q14) had low dup:del ratios (~ 1:1) and relatively low gene density, but they also had low values for both the duplication span and deletion span (≤10%; Additional file 1: Table S2). As such, low span values might represent cases in which the dup:del ratio alone is equivocal for interpreting novel microdeletions.

The above analyses suggest that outlying regions in the plot of gene density versus dup:del ratio are more likely to be pathogenic when deleted. To scrutinize this hypothesis, we tested its inverse, i.e., that deletion of non-outlying regions is benign. We observed multiple samples in our patient cohort with microdeletions ≥4 Mb, most of which we expected to be benign—or to have a mild or incompletely penetrant pathogenic phenotype—because of their presentation in expecting mothers. For all such microdeletions, we evaluated their respective gene densities, duplication spans, deletion spans, and dup:del ratios (yellow dots in Fig. 5e and Additional file 1: Figure S8; Additional file 2: Table S3). All but one of the regions directly supported our hypothesis because they were not outliers on either axis (Fig. 5e). We looked more deeply at the one variant that appeared to counter the hypothesis due to its very high dup:del ratio (yellow dot with arrow in Fig. 5e). Remarkably, this variant is a deletion of 13q34 that has recently been shown to be pathogenic, as it associates with intellectual disability and dysmorphism [23]. Therefore, rather than invalidate or weaken the hypothesis, the observed 13q34 microdeletion reinforces it.

Taken together, these observations suggest that parameterizing putative microdeletions on multiple biologically relevant axes, such as the two investigated here, could facilitate identification of pathogenic outliers and aid the clinical interpretation of novel fetal CNVs identified via NIPS.

## Discussion

Here we show that mCNVs are common on the chromosomes that NIPS interrogates (4.5% of patients have mCNV on chromosome 13, 18, or 21) and can cause frequent false positives if not properly neutralized at the algorithmic level. Even NIPS tests that share a common sequencing approach (e.g., WGS of cfDNA) may nevertheless have very different test specificities based on the sophistication of their mCNV handling. Using 87,255 empirical and 30,000 simulated samples, we quantified the impact on specificity of various mCNV-mitigation strategies and observed a very wide range of values. Our novel approach, which excludes bins in mCNVs from downstream calculations, reduces the expected rate of mCNV-caused false positives nearly 600-fold relative to the algorithms used in the early iterations of WGS-based NIPS and which may still be used in practice in clinical laboratories (1 in 520,000 vs. 1 in 860; Fig. 4). Finally, as a result of characterizing the frequency, length, and position of mCNVs, our work provides initial insight into the clinical interpretation of the novel fetal microdeletions that WGS-based NIPS can detect.

Algorithmic approaches tailored to mCNVs had better specificity than strategies that had robust features but were not mCNV-specific. For example, the value-filtering approach that excludes genomic bins based on their copy-number values (Fig. 4e) performed better than a method that simply used robust statistical metrics like the median and IQR (Fig. 4b). Value filtering has drawbacks, however, as the choice of threshold results in a tradeoff between specificity and sensitivity; a permissive threshold impairs specificity by retaining some bins from mCNVs, whereas an aggressive threshold lowers sensitivity by excluding bins that may not be in mCNVs. This tradeoff is avoided with an approach that identified the location of mCNVs and removed only the relevant bins from subsequent analysis. This mCNV filtering method had the highest specificity of the options considered, with a small *∆z*_*dup*_ in aggregate across all mCNV sizes, as well as low variance in the individual *∆z*_*dup*_ values (the z-score correction method was mCNV-aware but had high variance, which is expected to lower specificity).

Though mostly tailored to retain specificity, mCNV-mitigation approaches must also not reduce sensitivity for aneuploidies. Algorithms that retain all bins (“Simple” and “Robust”) were shown to be inferior due to their poor specificity, but they may have no net impact on sensitivity because they will have higher fetal-aneuploidy sensitivity in samples with maternal duplications and lower sensitivity in samples with maternal deletions. Strategies that remove outlying bins without directly identifying mCNVs (“Robust+Gaussian” and “Value filtering”) could slightly lower sensitivity for fetal aneuploidies (depending on the filtering cutoffs) because conservative filtering could superfluously remove bins not associated with mCNVs (Additional file 1: Figure S4). With the mCNV filtering approach, the small values and variance of *∆z*_*dup*_ mean that mCNVs minimally affect the z-score in either direction, suggesting that the filtering process does not compromise sensitivity. mCNV filtering could slightly boost sensitivity by avoiding false negative results in trisomic samples where the aneuploidy-inflated z-score is lowered to normal levels due to a maternal deletion.

While not directly investigated, mCNVs on non-tested chromosomes (i.e., autosomes other than chromosomes 13, 18, or 21)—or even mCNVs in other patient samples—could affect the z-score of a test chromosome [16]. WGS-based NIPS involves normalization of NGS read depth to calculate a z-score, and this normalization could include one or many chromosomes, as well as other samples in a background cohort. Robust normalization, including a large number of background samples and/or filtering out mCNVs before normalization, can mitigate spurious z-score changes due to cryptic mCNVs in the analysis pipeline.

Expert manual review of both z-scores and bin-level copy-number data across all autosomes can further safeguard against mCNV-caused false positives [24]. Based on our experience, strong collaboration between the manual reviewers and user-interface developers—as well as algorithmic flags that point out cases requiring careful scrutiny—can facilitate timely review at scale. However, we caution against an mCNV-mitigation strategy that relies solely on manual review of ideograms for putative positives [15] and foregoes a computational component that detects and assesses the impact of mCNVs. After all, most mCNVs do not cause false positives. Manual review without mCNV-specific algorithmic assistance could lower the sensitivity of the screen if trisomic samples with maternal duplications were dismissed as negatives. For instance, in addition to being cost-prohibitive and logistically challenging in a screening setting, a recently published recommendation [25] (currently used in practice [19]) supports dismissal of positive calls in samples that contain an mCNV verified by sequencing maternal white blood cell DNA. This guidance could decrease sensitivity relative to an mCNV-aware computational analysis that preserves true positive calls in aneuploid samples harboring mCNVs, where the mCNVs alone are insufficient to explain the observed z-scores.

Advances in WGS-based NIPS technology have enabled genome-wide microdeletion calling, but the challenge of interpreting positive findings could limit their clinical validity and utility. In principle, the clinical impact of a large fetal deletion stems from the cellular roles of its constituent genes and regulatory regions, but specific knowledge of these roles is often lacking. We identify the dup:del ratio as a general criterion that could advance the interpretation of large fetal CNVs; importantly, used together with gene density, each of the common microdeletions (plus the recently characterized 13q34 microdeletion [23]) was identified as an outlier. These bulk metrics might be well-suited for cases in which the genes encompassed by a novel microdeletion are not well-studied. We expect any information gained from the dup:del ratio to improve in quality with a larger patient cohort, as the observation of more mCNVs would enable use of a higher mCNV-count threshold for a bin to contribute to the duplication or deletion span. In addition, more examples of benign microdeletions observed in expecting mothers can further power the analysis.

## Conclusions

With proper algorithm design and extensive testing that leverages empirical and simulated data, high specificity in NIPS is possible even in the presence of mCNVs that range widely in size. Importantly, by using the mCNV-filtering approach described here, achieving robustness to mCNVs—and the corresponding rise in positive predictive value—does not compromise detection of true aneuploidies and, thereby, preserves both high sensitivity and a low test-failure rate. While the identification and analysis of mCNVs provide biological insight into the impact of large copy-number variants, mCNV removal upstream of fetal aneuploidy assessment is important to maintain exemplary test performance, which will be especially critical as NIPS adoption increases in the wider, general obstetric population.

## Additional files

Additional file 1: **Table S1.** Summary of the six algorithmic strategies tested. **Figure S1.** The desired versus observed mCNV size for simulations. **Figure S2.** Sensitivity of mCNV detection ascertained from simulations. **Figure S3.** Histogram of observed bin copy number estimates within mCNVs. **Figure S4.** Change in z-score due to mCNVs, the specificity attributable to false positives caused by duplications, and the proportion of available bins used for two cut-off options of the “Value filtering” method and the “mCNV filtering” method. **Figure S5.** Proportion of a chromosome covered by observed mCNVs. **Figure S6.** Change in z-score due to mCNVs and the specificity attributable to false positives caused by duplications: chromosome 13 as the basis for simulations. **Figure S7.** Change in z-score due to mCNVs and the specificity attributable to false positives caused by duplications: chromosome 18 as the basis for simulations. **Figure S8.** Duplication and deletion span values across all chromosomes. **Figure S9.** Varying the minimum required number of mCNV observations covering a genomic bin for that bin to count toward a duplication or deletion span. **Figure S10.** Bootstrapping analysis of duplication and deletion spans. **Table S2.** Properties of ICCG microdeletions and identified maternal deletions greater than 4 Mb. (PDF 14030 kb)

Additional file 2: **Table S3.** Identified autosomal maternal CNVs. (CSV 13077 kb)

## Abbreviations

cfDNA: cell-free DNA
CNV: Copy-number variant
HIPAA: Health Insurance Portability and Accountability Act
ICCG: International Collaboration for Clinical Genomics (formerly ISCA)
IQR: Interquartile range
kb: kilobases
Mb: Megabases
mCNV: maternal copy-number variant
NIPS: Noninvasive prenatal screening
WGS: Whole-genome sequencing

## References

[1] Norton ME, Jacobsson B, Swamy GK, Laurent LC, Ranzini AC, Brar H (2015). Cell-free DNA analysis for noninvasive examination of trisomy. N Engl J Med.

[2] Gregg AR, Skotko BG, Benkendorf JL, Monaghan KG, Bajaj K, Best RG (2016). Noninvasive prenatal screening for fetal aneuploidy, 2016 update: a position statement of the American College of Medical Genetics and Genomics. Genet Med.

[3] ACOG. Cell-free DNA screening for fetal aneuploidy. Committee Opinion No. 640. Obstet Gynecol [Internet]. 2015;126. Available from: https://journals.lww.com/greenjournal/fulltext/2015/09000/Committee_Opinion_No__640___Cell_Free_Dna.51.aspx. Accessed 8 Oct 2018. 10.1097/AOG.0000000000001051 26287791

[4] Akolekar R, Beta J, Picciarelli G. Procedure-related risk of miscarriage following amniocentesis and chorionic villus sampling: a systematic review and meta-analysis. Ultrasound Obstet Gynecol [Internet]. Wiley Online Library; 2015; Available from: http://onlinelibrary.wiley.com/doi/10.1002/uog.14636/full. Accessed 8 Oct 2018. 10.1002/uog.14636 25042845

[5] Snyder MW, Simmons LE, Kitzman JO, Coe BP, Henson JM, Daza RM (2015). Copy-Number Variation and False Positive Prenatal Aneuploidy Screening Results. N Engl J Med.

[6] Strom CM, Maxwell MD, Owen R (2017). Improving the Accuracy of Prenatal Screening with DNA Copy-Number Analysis. N Engl J Med.

[7] Chudova DI, Sehnert AJ, Bianchi DW (2016). Copy-Number Variation and False Positive Prenatal Screening Results. N Engl J Med.

[8] Hartwig TS, Ambye L, Sørensen S, Jørgensen FS (2017). Discordant non-invasive prenatal testing (NIPT) - a systematic review. Prenat Diagn.

[9] Srinivasan A, Bianchi DW, Huang H, Sehnert AJ, Rava RP (2013). Noninvasive detection of fetal subchromosome abnormalities via deep sequencing of maternal plasma. Am J Hum Genet.

[10] Ehrich M, Tynan J, Mazloom A, Almasri E, McCullough R, Boomer T (2017). Genome-wide cfDNA screening: clinical laboratory experience with the first 10,000 cases. Genet Med.

[11] Benjamini Y, Speed TP (2012). Summarizing and correcting the GC content bias in high-throughput sequencing. Nucleic Acids Res.

[12] Chandrananda D, Thorne NP, Ganesamoorthy D, Bruno DL, Benjamini Y, Speed TP (2014). Investigating and correcting plasma DNA sequencing coverage bias to enhance aneuploidy discovery. PLoS One.

[13] Chiu RWK, Chan KCA, Gao Y, Lau VYM, Zheng W, Leung TY (2008). Noninvasive prenatal diagnosis of fetal chromosomal aneuploidy by massively parallel genomic sequencing of DNA in maternal plasma. Proc Natl Acad Sci U S A.

[14] Wan X, Wang W, Liu J, Tong T (2014). Estimating the sample mean and standard deviation from the sample size, median, range and/or interquartile range. BMC Med Res Methodol.

[15] Strom CM, Anderson B, Tsao D, Zhang K, Liu Y, Livingston K (2017). Improving the Positive Predictive Value of Non-Invasive Prenatal Screening (NIPS). PLoS One.

[16] van den Boom D, Ehrich M, Kim SK (2015). Copy-Number Variation and False Positive Results of Prenatal Screening. N Engl J Med.

[17] Sparks AB, Wang ET, Struble CA, Barrett W, Stokowski R, McBride C (2012). Selective analysis of cell-free DNA in maternal blood for evaluation of fetal trisomy. Prenat Diagn.

[18] Kingsley C, Wang E, Oliphant A (2015). Commentary: Copy-Number Variation and False Positive Results of Prenatal Screening. N Engl J Med.

[19] Jiang F, Ren J, Chen F, Zhou Y, Xie J, Dan S (2012). Noninvasive Fetal Trisomy (NIFTY) test: an advanced noninvasive prenatal diagnosis methodology for fetal autosomal and sex chromosomal aneuploidies. BMC Med Genet.

[20] Fan HC, Quake SR (2010). Sensitivity of noninvasive prenatal detection of fetal aneuploidy from maternal plasma using shotgun sequencing is limited only by counting statistics. PLoS One.

[21] Fu W, Zhang F, Wang Y, Gu X, Jin L (2010). Identification of copy number variation hotspots in human populations. Am J Hum Genet.

[22] Clinical Genome Resource. ISCA Curated Pathogenic/Benign Regions [Internet]. ClinGen Clinical Genome Resource. Available from: https://www.clinicalgenome.org/toolkits/array-analysis-toolkit/. Cited 31 Dec 2017.

[23] Reinstein E, Liberman M, Feingold-Zadok M, Tenne T, Graham JM (2016). Terminal microdeletions of 13q34 chromosome region in patients with intellectual disability: Delineation of an emerging new microdeletion syndrome. Mol Genet Metab.

[24] Bayindir B, Dehaspe L, Brison N, Brady P, Ardui S, Kammoun M (2015). Noninvasive prenatal testing using a novel analysis pipeline to screen for all autosomal fetal aneuploidies improves pregnancy management. Eur J Hum Genet.

[25] Zhou X, Sui L, Xu Y, Song Y, Qi Q, Zhang J (2017). Contribution of maternal copy number variations to false-positive fetal trisomies detected by noninvasive prenatal testing. Prenat Diagn.

