# Supplementary material for: Strategies to minimize false positives and interpret novel microdeletions based on maternal copy-number variants in 87,000 noninvasive prenatal screens
Source: BMC Med Genomics. 2018 Oct 19;11:90. doi: 10.1186/s12920-018-0410-6 (PMC6194617; doi:10.1186/s12920-018-0410-6)
Supplement: Supplementary file 1 — Table S1. Summary of the six algorithmic strategies tested. Figure S1. The desired versus observed mCNV size for simulations. Figure S2. Sensitivity of mCNV detection ascertained from simulations. Figure S3. Histogram of observed bin copy number estimates within mCNVs. Figure S4. Change in z-score due to mCNVs, the specificity attributable to false positives caused by duplications, and the proportion of available bins used for two cut-off options of the “Value filtering” method and the “mCNV filtering” method. Figure S5. Proportion of a chromosome covered by observed mCNVs. Figure S6. Change in z-score due to mCNVs and the specificity attributable to false positives caused by duplications: chromosome 13 as the basis for simulations. Figure S7. Change in z-score due to mCNVs and the specificity attributable to false positives caused by duplications: chromosome 18 as the basis for simulations. Figure S8. Duplication and deletion span values across all chromosomes. Figure S9. Varying the minimum required number of mCNV observations covering a genomic bin for that bin to count toward a duplication or deletion span. Figure S10. Bootstrapping analysis of duplication and deletion spans. Table S2. Properties of ICCG microdeletions and identified maternal deletions greater than 4 Mb. (PDF 14030 kb) [file 12920_2018_410_MOESM1_ESM.pdf]

Strategy name	*Measure of central tendency*	*Measure of dispersion*	*Outlier exclusions / Notes*
Simple	Mean	Raw standard deviation	None
Robust	Median	Standard deviation estimated from IQR	None
Robust+Gaussian	Median	Standard deviation estimated from IQR	Excludes bin copy-number values more than four standard deviation from a Gaussian fit
Z-correction	Median	Standard deviation estimated from IQR	Corrects z-score using a size- and chromosome-specific offset based on simulations
Value filtering	Median	Standard deviation estimated from IQR	Excludes bin copy-number values less than 1.5 or more than 2.5
mCNV filtering	Median	Standard deviation estimated from IQR	Excludes bins determined to be within an mCNV

Table S1: Summary of the six algorithmic strategies tested. IQR: interquartile range.

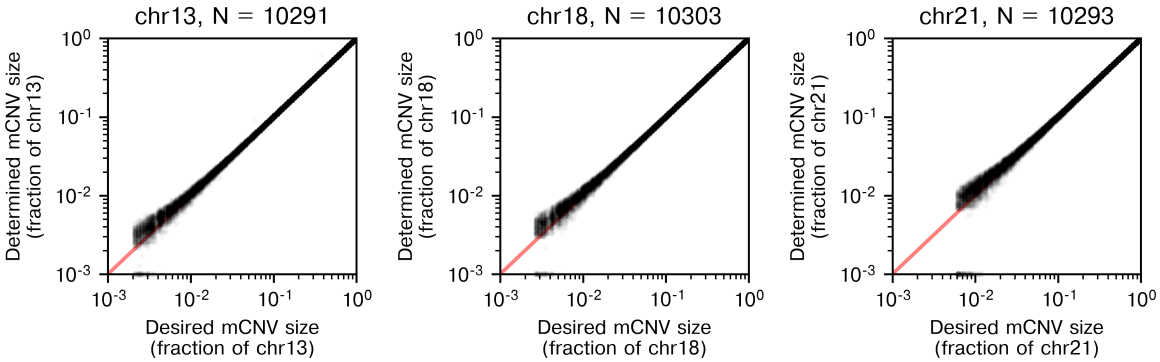

Figure S1: The desired versus observed mCNV size (fraction of chromosome) for simulations. The desired size was used in analyses. If a simulated mCNV was not detected, it is plotted at 10^{-3} (y-axis) in this plot for visualization purposes.

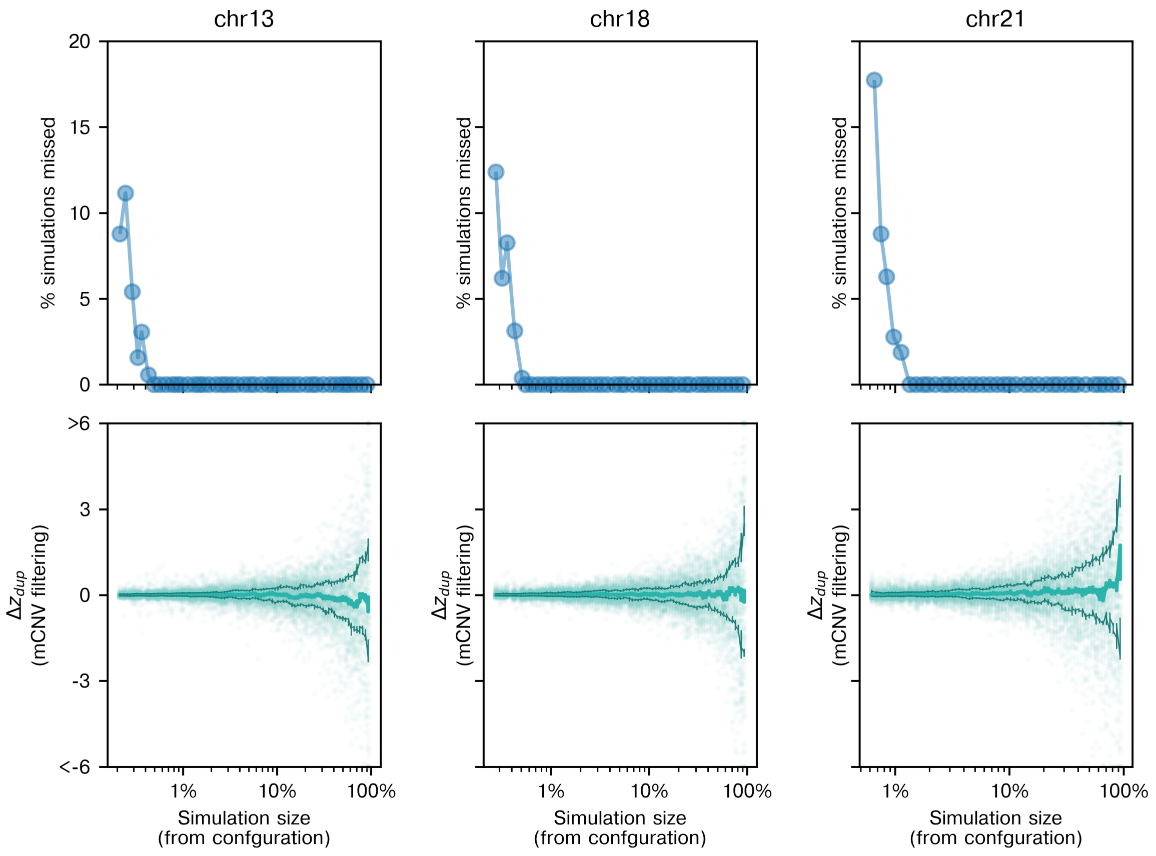

Figure S2: Sensitivity of mCNV detection ascertained from simulations. Top row: the percentage of simulations missed based on the desired simulation size. Bottom row: the effect of simulated mCNVs on the aneuploidy z-score for the “mCNV filtering” strategy (summarizing results also shown in Figure 4F, Figure S3C, Figure S5, Figure S6).

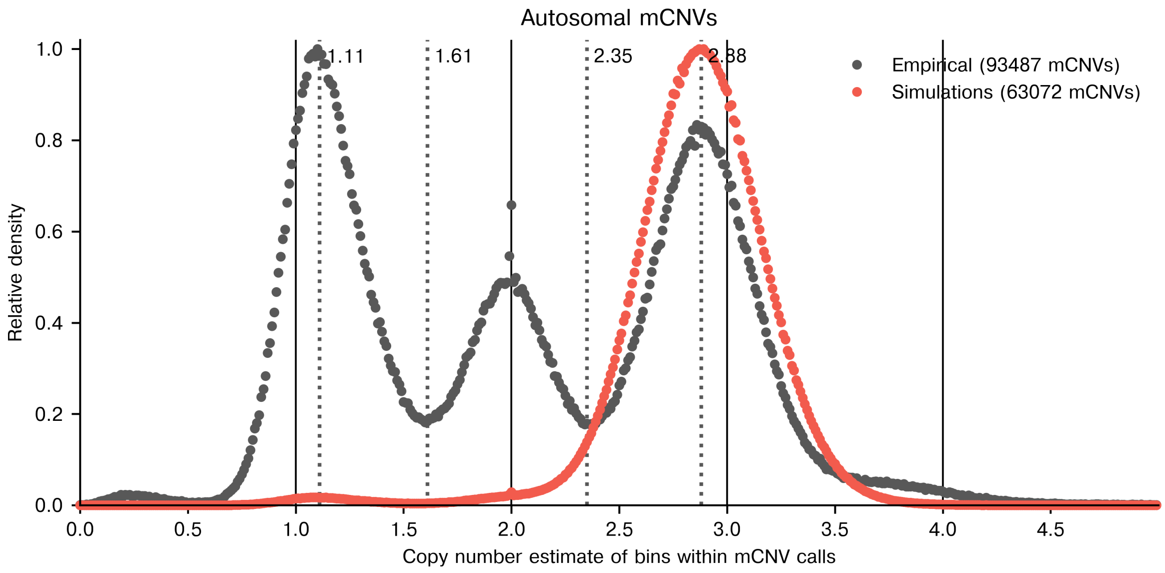

Figure S3: Histogram of observed bin copy number estimates within mCNVs. Empirical mCNV calls include some normal bins (peak around copy number 2). Simulations were generated to matched the observed peak at 2.88 rather than 3.00 for duplications. The "Value filtering" approach could filter bins if their estimated copy number is outside the expected normal range of 1.5-2.5, or the empirically observed normal range of 1.61-2.35.

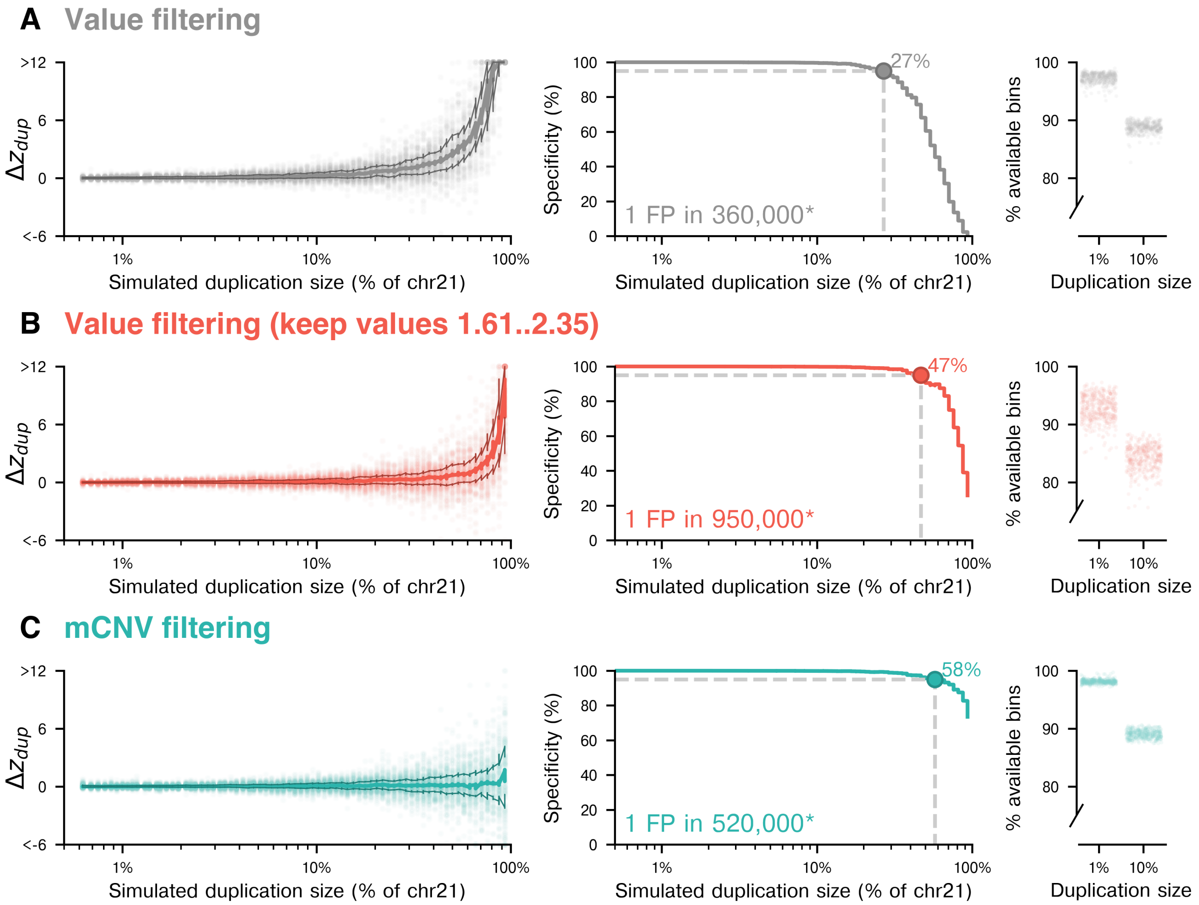

Figure S4: Change in z-score due to mCNVs (leftmost column), the specificity attributable to false positives caused by duplications (middle column), and the proportion of available bins used (rightmost column) for two cut-off options of the "Value filtering" method and the "mCNV filtering" method. The estimate for the false positive rate is based on all chromosomes; chromosome 21 data is used elsewhere in the figure. In the rightmost column, the proportion of available bins was calculated for mCNVs around 1% and 10% of chromosome 21 to illustrate its dependence on mCNV size. **(A)** Using cut-offs of $c_{low} = 1.5$ and $c_{high} = 2.5$ results in higher false positive rate than the mCNV filtering approach. **(B)** Using cut-offs of $c_{low} = 1.61$ and $c_{high} = 2.35$ results in an estimated lower mCNV-attributable false positive rate, but discards more data than the "mCNV filtering" approach, in addition to being severely affected by large mCNVs. **(C)** The mCNV filtering approach has high specificity and low variance in the number of bins that are filtered.

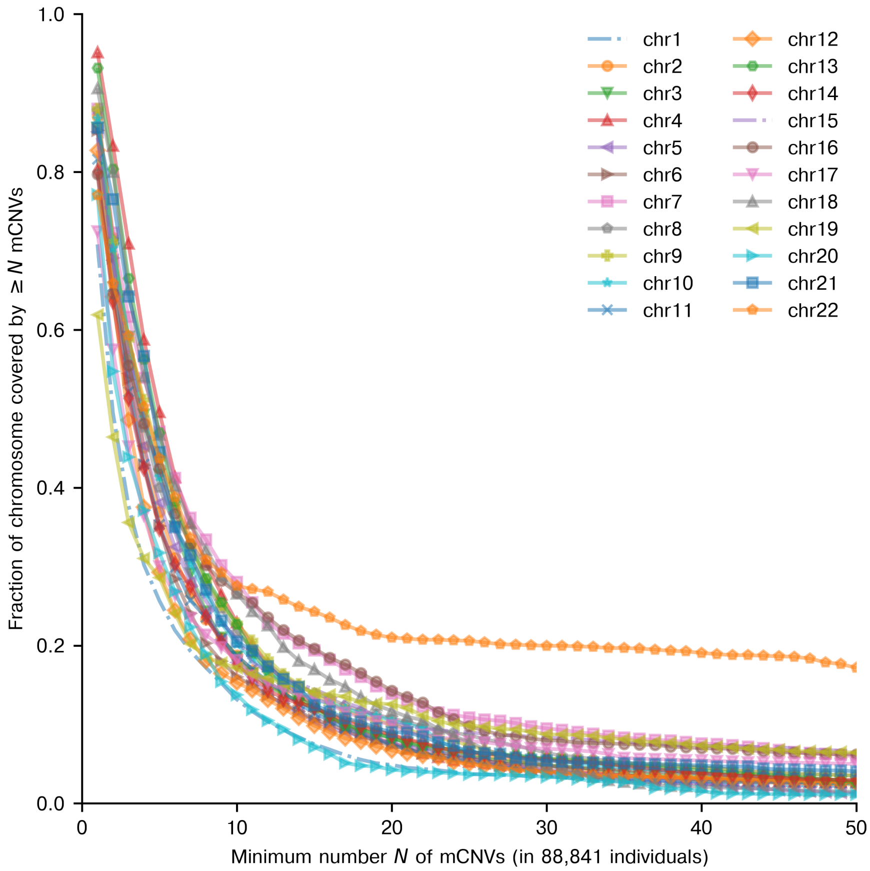

Figure S5: Proportion of a chromosome (mappable regions only) covered by mCNVs observed in the dataset of 88,841 NIPS samples.

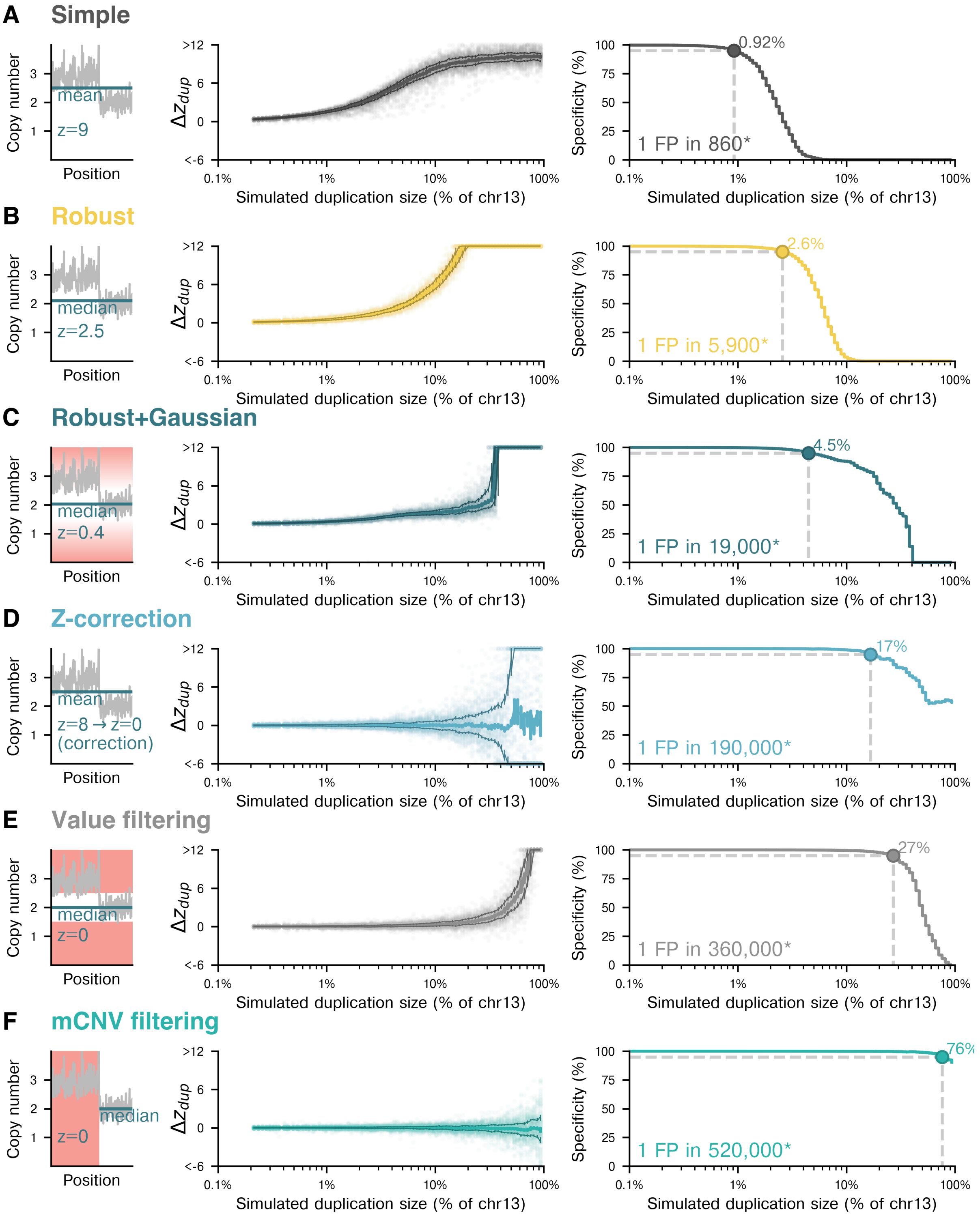

Figure S6: Change in z-score due to mCNVs and the specificity attributable to false positives caused by duplications: chromosome 13 as the basis for simulations. (*) Estimate for false positives includes all chromosomes.

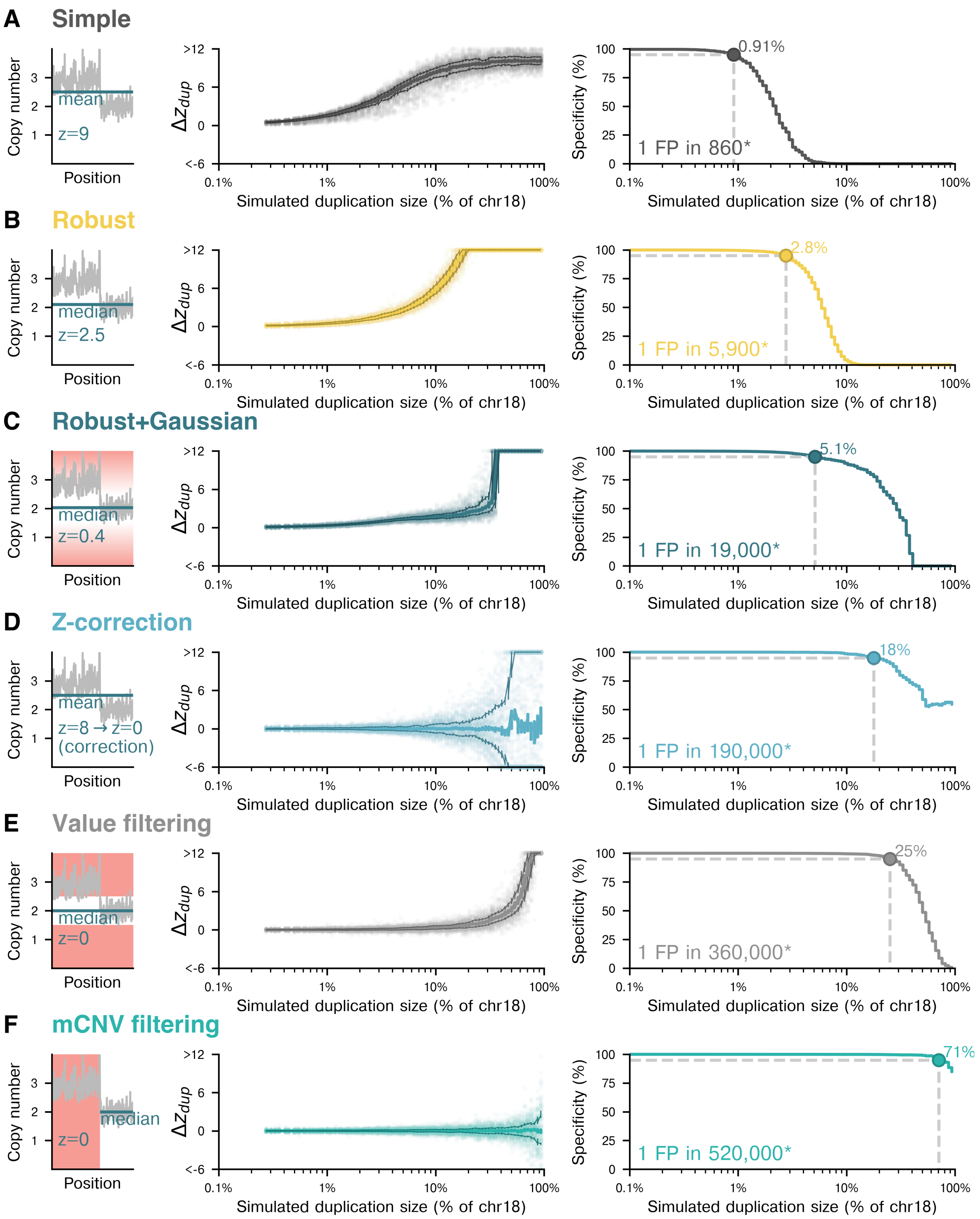

Figure S7: Change in z-score due to mCNVs and the specificity attributable to false positives caused by duplications: chromosome 18 as the basis for simulations. (*) Estimate for false positives includes all chromosomes.

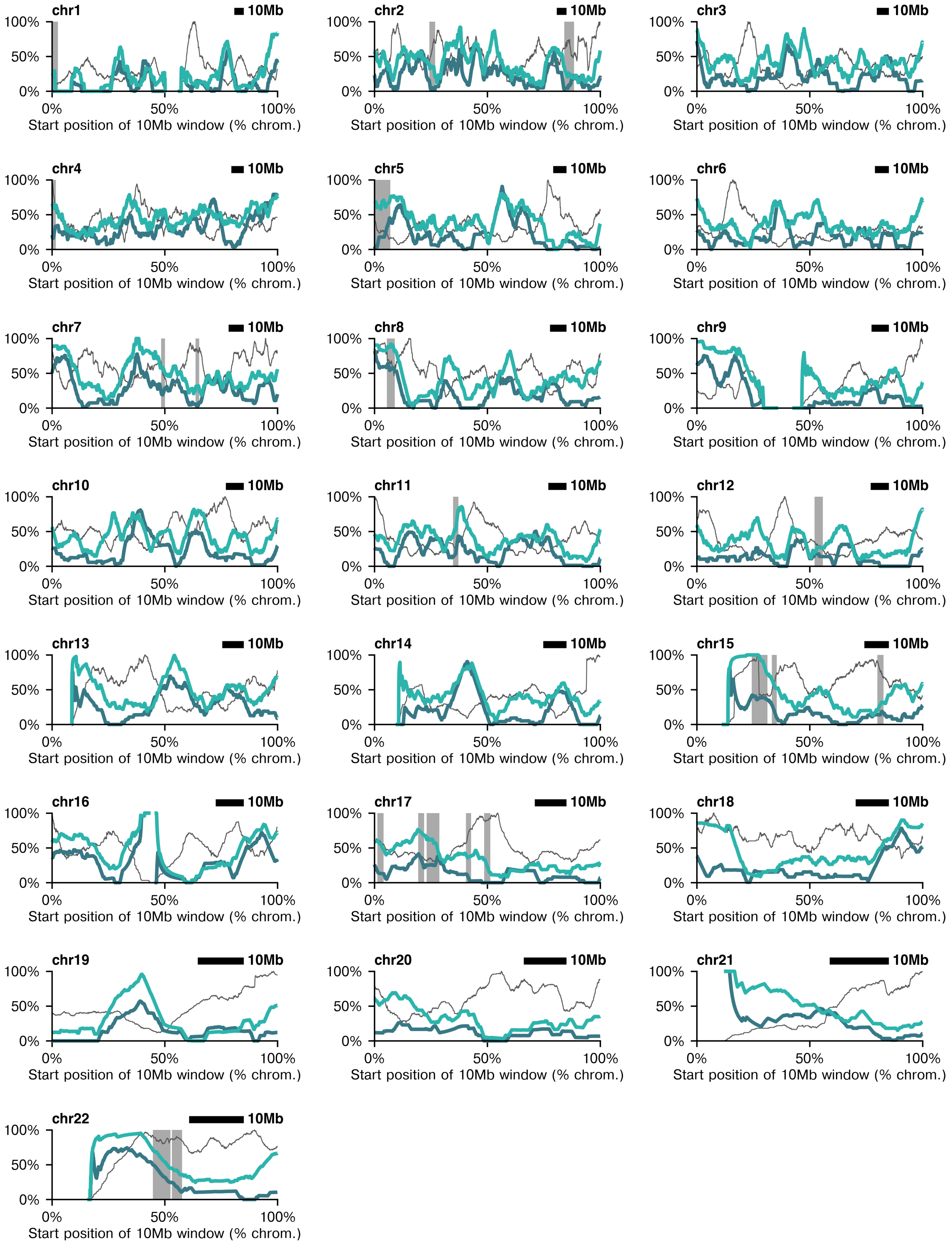

Figure S8: **Duplication (teal)** and **deletion (dark blue)** span values across all chromosomes; gene content (gray), normalized within the chromosome. The mCNV span indicates the proportion of a 10Mb window that has at least 3 mCNVs covering it. Gray background indicates pathogenic ICCG microdeletion regions.

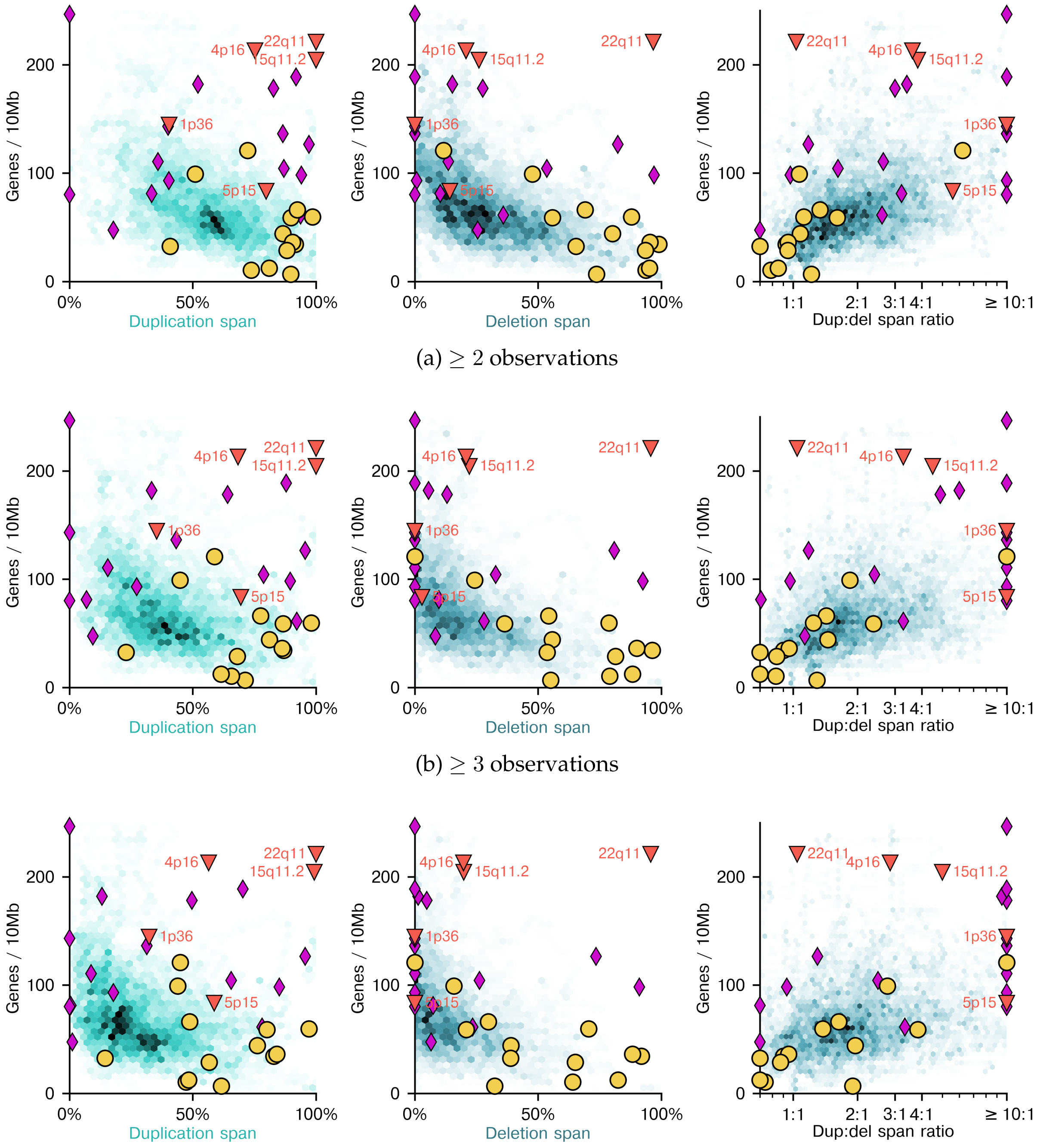

(a) ≥ 2 observations

(b) ≥ 3 observations

(c) ≥ 4 observations

Figure S9: Varying the minimum required number of mCNV observations covering a genomic bin for that bin to count toward a duplication or deletion span. A minimum of 3 mCNVs in the dataset of 88,841 patients was used in the manuscript. Red annotated triangles: common pathogenic ICCG microdeletions; purple diamonds: other pathogenic ICCG variants; yellow dots: observed mCNVs (at least 4Mb).

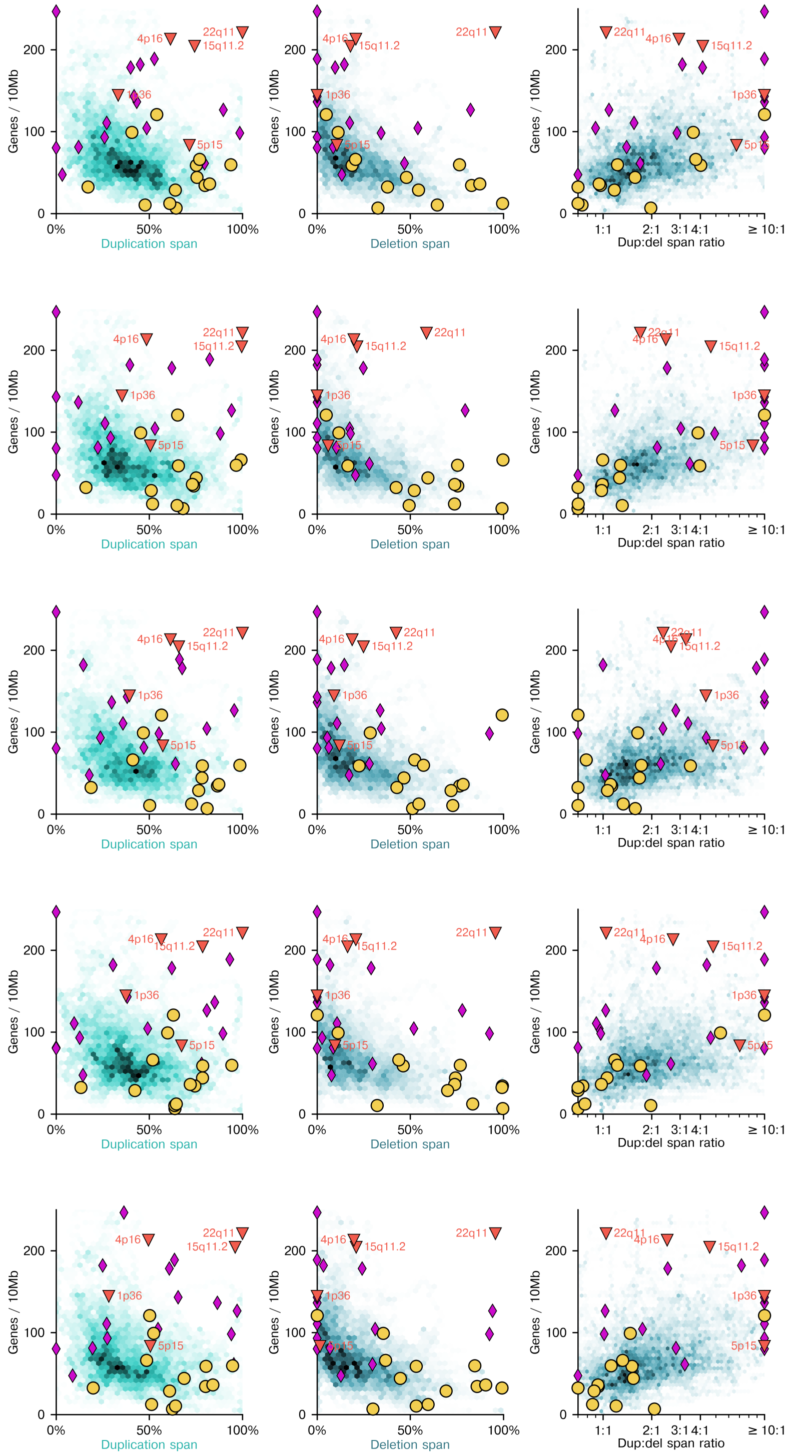

Figure S10: Bootstrapping analysis of duplication and deletion spans. Bootstrapping performed by choosing samples with replacement with five different pseudo-random number generator seeds. Red annotated triangles: common pathogenic ICCG variants; purple diamonds: other pathogenic ICCG variants; yellow dots: observed mCNVs (at least 4Mb).

Name	*Position*	*Size*	*Dup:del span*	*Percentile*	*Genes per 10Mb*	*Percentile*	*Essential genes*
5p15 microdeletion (Cri-du-chat)	chr5:37693-11347262	11.1Mb	0.69:0.03 $\approx$ 24.06	99%	83.1	71%	4
15q11.2 microdeletion (Prader-Willi/Angelman class 1)	chr15:22876632-28557186	5.1Mb	1.00:0.22 $\approx$ 4.51	86%	204.2	99%	2
4p16 microdeletion (Wolf-Hirschhorn)	chr4:72448-2327204	2.0Mb	0.68:0.21 $\approx$ 3.29	76%	212.9	99%	4
1p36 microdeletion	chr1:834083-5408761	2.0Mb	0.35:0.00 $\approx \infty$	100%	144.3	95%	1
22q11 microdeletion	chr22:18661725-21561514	1.8Mb	1.00:0.96 $\approx$ 1.05	10%	220.7	99%	7
2q33.1 microdeletion	chr2:196925089-205206940	8.1Mb	0.27:0.00 $\approx \infty$	100%	93.0	78%	6
8p23.1 microdeletion	chr8:8119295-11765719	3.7Mb	0.79:0.33 $\approx$ 2.40	62%	104.2	85%	0
2p15p16.1 microdeletion	chr2:57741796-61738334	3.6Mb	0.09:0.08 $\approx$ 1.13	13%	47.5	26%	1
12q14 microdeletion	chr12:65071919-68645525	3.5Mb	0.07:0.10 $\approx$ 0.71	2%	81.2	69%	0
2q37 microdeletion	chr2:239954693-242930600	2.9Mb	0.64:0.13 $\approx$ 4.89	88%	178.1	98%	2
Smith-Magenis	chr17:16757111-20219651	2.9Mb	0.33:0.06 $\approx$ 6.00	91%	181.9	98%	3
Potocki-Shaffer	chr11:43985277-46064560	2.1Mb	0.16:0.00 $\approx \infty$	100%	110.6	87%	1
3q29 microdeletion	chr3:195756054-197344665	1.5Mb	0.88:0.00 $\approx \infty$	100%	188.8	98%	2
22q11.2 distal microdeletion	chr22:22115848-23696229	1.4Mb	0.96:0.81 $\approx$ 1.18	15%	126.6	92%	0
Hereditary neuropathy with liability to pressure palsies	chr17:14097914-15422955	1.3Mb	0.90:0.93 $\approx$ 0.97	7%	98.1	82%	1
17q12 microdeletion	chr17:34856056-36248918	1.3Mb	0.43:0.00 $\approx \infty$	100%	136.4	94%	3
15q24 microdeletion	chr15:74377174-76162277	1.3Mb	0.00:0.00 $\approx \infty$	100%	246.5	100%	3
15q13.3 microdeletion (BP4-5)	chr15:31137104-32445408	1.3Mb	0.92:0.28 $\approx$ 3.28	75%	61.1	46%	0
Split hand/foot malformation 1 (SHFM1)	chr7:95533860-96779486	1.3Mb	0.00:0.00 $\approx \infty$	100%	80.3	68%	0
Williams-Beuren	chr7:72744454-74142513	1.1Mb	0.00:0.00 $\approx \infty$	100%	143.1	95%	2
13.5Mb 18q22.1-18q22.2 maternal deletion	chr18:64240000-78020000	13.4Mb	0.81:0.56 $\approx$ 1.46	29%	44.3	22%	4
9.8Mb 3p12.1-3p12.2 maternal deletion	chr3:73980000-84460000	9.9Mb	0.71:0.55 $\approx$ 1.29	22%	6.7	0%	0
8.2Mb 18p11.22-18p11.23 maternal deletion	chr18:160000-8660000	8.2Mb	0.87:0.36 $\approx$ 2.38	61%	58.8	41%	2
7.3Mb 10q22.3-10q23.1 maternal deletion	chr10:81560000-88960000	7.3Mb	0.78:0.54 $\approx$ 1.43	28%	66.2	53%	0
5.9Mb 3p26.1-3p26.2 maternal deletion	chr3:220000-6340000	5.9Mb	0.87:0.96 $\approx$ 0.90	5%	34.3	10%	0
5.6Mb 3p26.1-3p26.2 maternal deletion	chr3:240000-6040000	5.6Mb	0.86:0.90 $\approx$ 0.96	7%	36.2	12%	0
5.3Mb 13q33.3-13q34 maternal deletion	chr13:109160000-115120000	5.3Mb	0.59:0.00 $\approx \infty$	100%	120.8	90%	4
5.2Mb 7p21.2-7p21.3 maternal deletion	chr7:11260000-16500000	5.3Mb	0.68:0.81 $\approx$ 0.84	3%	28.6	6%	0
5.2Mb 12q21.31-12q21.32 maternal deletion	chr12:85340000-90560000	5.1Mb	0.23:0.54 $\approx$ 0.43	0%	32.6	9%	0
4.8Mb 3p12.1-3p12.2 maternal deletion	chr3:80620000-85400000	4.8Mb	0.66:0.79 $\approx$ 0.83	3%	10.5	1%	0
4.3Mb 13q14.11-13q14.12 maternal deletion	chr13:41820000-46160000	4.3Mb	0.45:0.24 $\approx$ 1.85	46%	99.1	82%	2
4.1Mb 4q35.1-4q35.2 maternal deletion	chr4:186080000-190280000	4.1Mb	0.98:0.79 $\approx$ 1.25	19%	59.5	43%	0
4.1Mb 4q34.3 maternal deletion	chr4:178040000-182120000	4.1Mb	0.61:0.88 $\approx$ 0.70	2%	12.3	1%	0

Table S2: Properties of ICCG microdeletions (first two sections: pathogenic, commonly tested pathogenic), and identified maternal deletions greater than 4Mb (third section). The size is determined by the mappable portion of the region. The percentile columns are in relation to 10Mb autosomal moving windows. List of 1,580 essential genes obtained from PMID 26627737.
